# Transformation-Guided Genome Mining Provides Access to Brominated Lanthipeptides

**DOI:** 10.1021/acs.orglett.4c04529

**Published:** 2025-01-17

**Authors:** Nirmal Saha, F. N. U. Vidya, Youran Luo, Wilfred A. van der Donk, Vinayak Agarwal

**Affiliations:** † School of Chemistry and Biochemistry, Georgia Institute of Technology, Atlanta, Georgia 30332, United States; ‡ Department of Chemistry, University of Illinois at Urbana−Champaign, Urbana, Illinois 61801, United States; § Carl R. Woese Institute for Genomic Biology, University of Illinois at Urbana−Champaign, Urbana, Illinois 61801, United States; ∥ Howard Hughes Medical Institute, University of Illinois at Urbana−Champaign, Urbana, Illinois 61801, United States; ⊥ School of Biological Sciences, Georgia Institute of Technology, Atlanta, Georgia 30332, United States

## Abstract

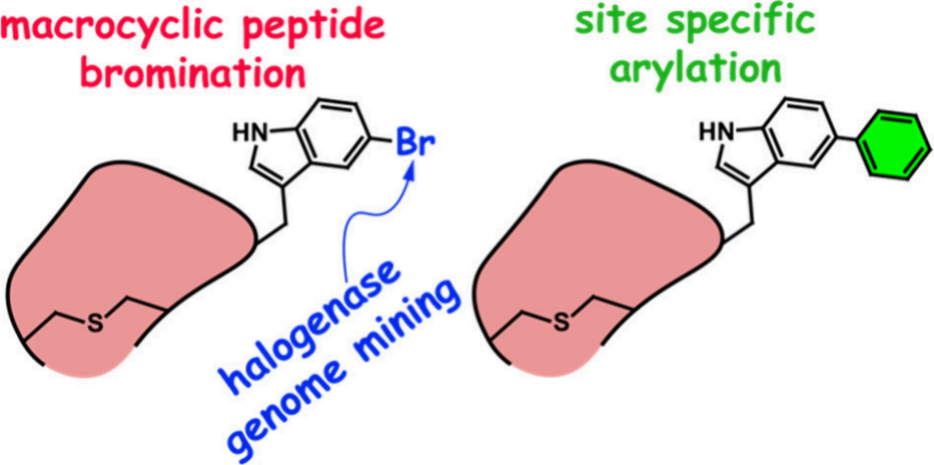

Natural product biosynthesis is nature’s tinkering ground for developing new enzymes that can achieve chemical transformations that are outside the purview of traditional chemical catalysis. Herein we describe a genome mining approach that leads to the discovery of a halogenase that regioselectively brominates a tryptophan side chain indole for a macrocyclic peptide substrate, enabling downstream chemical arylation by Suzuki–Miyaura coupling. The halogenase was found to prefer a macrocyclic peptide substrate over a linear peptide. The brominase presents a starting point for biocatalytic access to macrocyclic peptides bearing a chemically versatile aryl-bromide reactive handle

There is a resurgence of intense pharmacological interest in macrocyclic peptides; they are the Goldilocks molecules bridging selective but difficult to maneuver biologics and small molecules that are easier to deliver but struggle in engaging large biomolecular surfaces. Macrocyclization endows peptides with membrane permeability and stability against proteolysis.^1−3^ Among the macrocyclization modalities, intramolecular thioether bonds are well represented among bioactive peptides.^4^ Lanthipeptides are ribosomally derived and post-translationally modified peptides (RiPPs) that are macrocyclized by thioether bond formation between a Cys thiol and a Cβ carbon atom of dehydrated Ser and Thr side chains. Chemoenzymatic derivatization of lanthipeptides is the key to fully exploiting their pharmacological potential.

Akin to the prominence of brominated intermediates in organic synthesis, enzymatic bromination of ribosomally derived peptides and proteins allows for their chemical derivatization by transition-metal-assisted C–C bond-forming reactions.^5−7^ Enzymatic bromination benefits from the regiospecific delivery of the bromine atom onto the peptide. With a view toward downstream chemical derivatization, bromination is particularly desirable. While not suffering from the instability of aryl iodides, the increased reactivity of aryl bromides as compared to that of aryl chlorides allows for C–C bond-forming reactions to proceed in aqueous solvents under mild reaction conditions. Considering these observations, in this study, we adopted a genome mining strategy directed toward the discovery of a biosynthetic gene cluster (BGC) that could furnish macrocyclic lanthipeptides bearing an aryl bromide reactive handle. We further explored the transition-metal-assisted chemical derivatization of macrocyclic RiPPs.

We have previously described the activity of the flavin-dependent halogenase SrpI that brominates a tryptophan indole of linear peptides.^8^ Using SrpI as a query sequence, we mined for halogenases in sequence databases. The ensuing hits were organized into a sequence similarity network (SSN) illustrated in Figure 1A.^9^ With the motivation to access macrocyclic halogenated peptides, we mined this SSN for BGCs that possess a SrpI-like halogenase encoding gene in the neighborhood of a lanthionine synthetase (LanM) encoding gene. LanMs catalyze macrocyclizing thioether bond formation using the above-mentioned strategy of ATP-dependent Ser/Thr side chain dehydration and the addition of a Cys side chain thiol at the Cβ of the dehydroalanine/dehydrobutyrine (Dha/Dhb) residue.^10^ Using this strategy, four BGCs were detected wherein an SrpI-like halogenase was encoded in the vicinity of a LanM (Figure S1). To bias the search for the discovery of brominases that do not suffer from contaminating chlorination activity, we chose the marine cyanobacterium *Moorena producens*-derived RiPP BGC (henceforth abbreviated as the *mpp* BGC) for further experimental evaluation; marine cyanobacterial halogenases have a propensity to be obligate brominases.^11,12^

**Figure 1:**
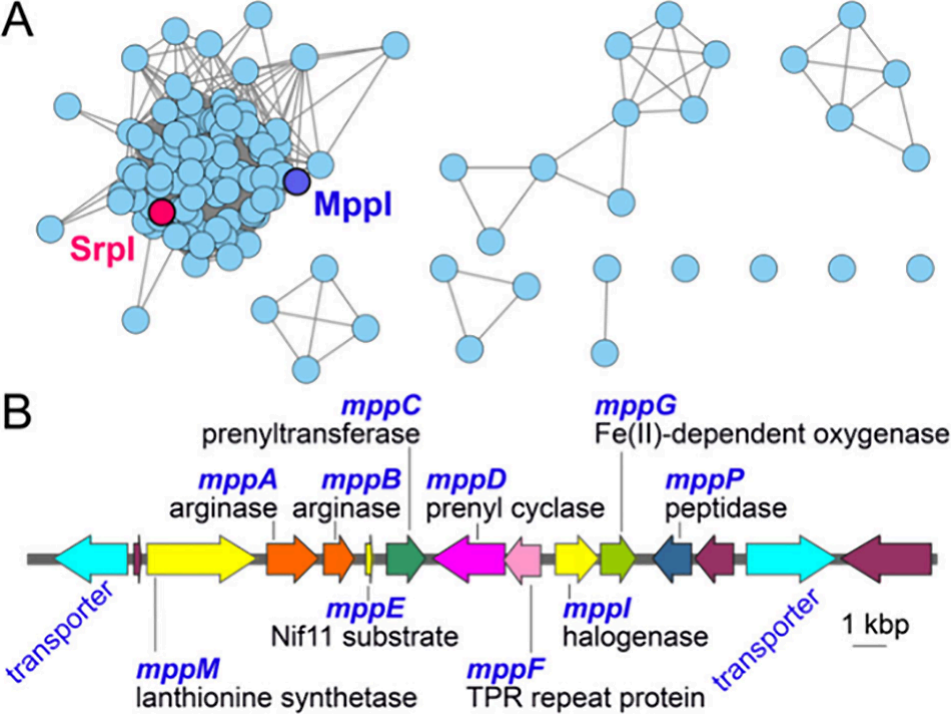
(A) SSN with 124 sequences generated using SrpI as the query demonstrating the clustering of nodes corresponding to SrpI and MppI halogenases. (B) The mpp BGC with predicted activities of encoded enzymes annotated. Genes colored purple are hypothetical genes for which we could not predict activity or function.

In addition to the halogenase and LanM encoding genes *mppI* and *mppM*, respectively, the *mpp* BGC possesses genes encoding the RiPP precursor peptide (*mppE*), two genes encoding arginases (*mppA* and *mppB*), a prenyltransferase and a terpene synthase (*mppC* and *mppD*, respectively), and an oxygenase (*mppG*) (Figure 1B). RiPP precursor peptides comprise an N-terminal leader and a C-terminal core; the leader guides the post-translational modification of the core peptide. Consistent with this architecture, the MppE precursor peptide possess a Nif11-like 77 amino acid long leader appended to a 7 amino acid ACWRWSG core (Table S1).^13^ Eventual proteolytic release of the modified core is postulated to be catalyzed by a peptidase encoded by the *mppP* gene; even though the MppE leader terminates in a Gly-Gly motif, there is no peptidase domain embedded within either of the two transporters encoded in the vicinity of the *mpp* BGC (Figure 1B).^14^ The RiPPs encoded by the *mpp* BGC are as yet cryptic; this study is directed toward describing the activity of the MppI halogenase in conjunction with the macrocyclizing lanthionine synthetase MppM.

Coexpression of the precursor peptide encoding gene *mppE* together with the LanM encoding gene *mppM* in *Escherichia coli* led to lanthionine ring formation between the MppE Cys79 and Ser83 side chains (Figure 2, Figures S2–S4). Degradation experiments established the configuration of the lanthionine ring as dl-lanthionine (Figure S5).^15^ When *mppE* was coexpressed with *mppM* and *mppI*, we observed the product mass corresponding to the addition of a bromine atom upon the macrocyclic RiPP; no chlorination was observed (Figure 2, Figure S6). Flavin-dependent halogenases bear a conserved catalytic Lys residue in their active sites that is implicated in bridging the site for halide oxidation proximal to the flavin cofactor and the site where the aryl substrate is bound.^16−18^ Consistent with this mechanistic hypothesis, the MppI Lys73 → Ala mutation abolished bromination (Figure S7).

**Figure 2:**
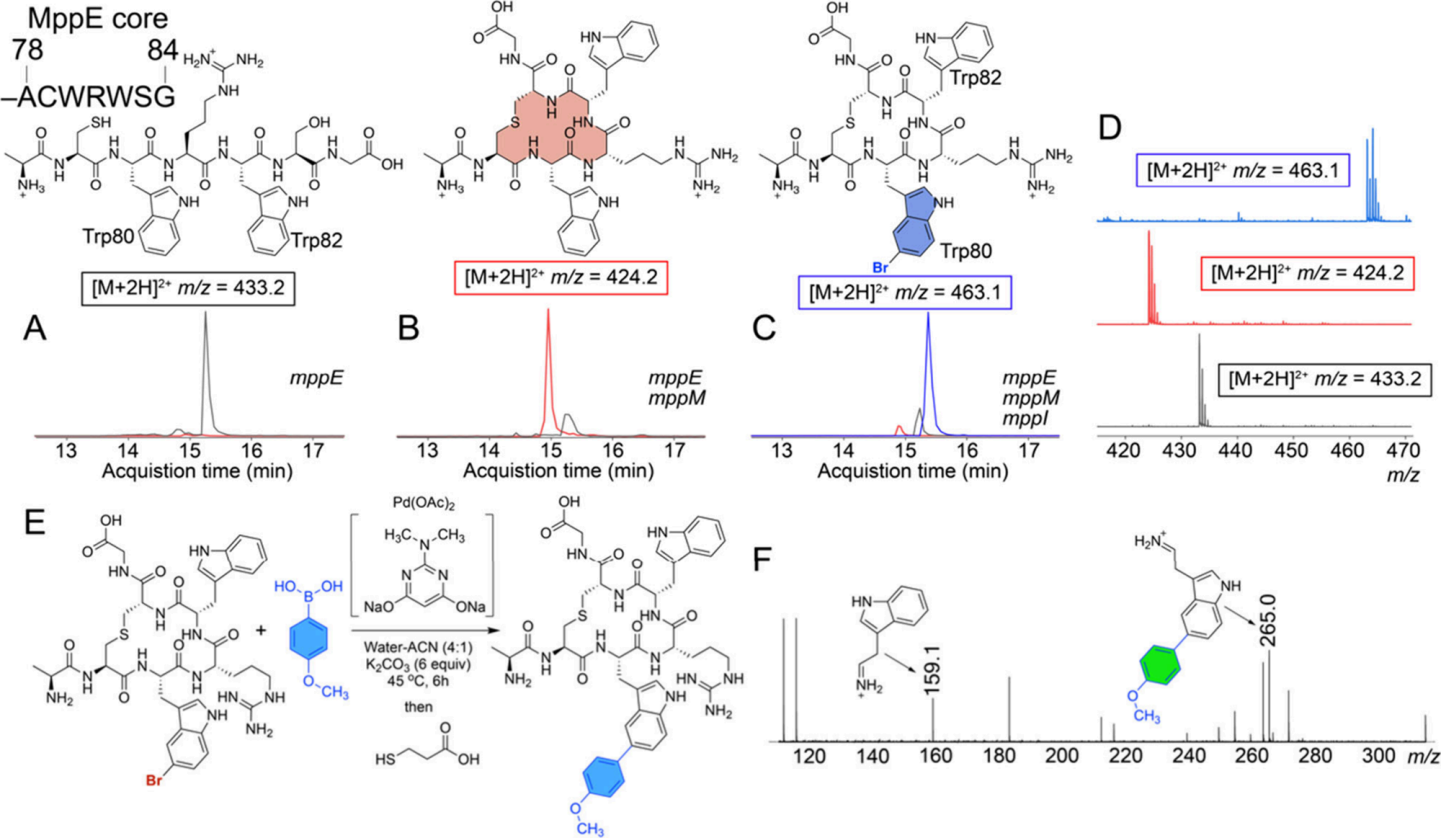
Extracted ion chromatograms (EICs) in black, red, and blue, respectively, demonstrating the presence of linear MppE, macrocyclized MppE, and macrocyclized and brominated MppE core peptides when (A) *mppE* was expressed in *E. coli*, (B) *mppE* was coexpressed with *mppM*, and (C) *mppE* was coexpressed with *mppM* and *mppI*. The EICs were generated by using the [M + 2H]^2+^ ions. The MppE core was excised from the leader using the LahT150 protease.^14^ (D) [M + 2H]^2+^ MS^1^ spectra for (from bottom to top) the linear, macrocyclized, and macrocyclized and brominated MppE core peptide. (E) Scheme for SMCC of 4-methoxyphenyl boronic acid to the brominated lanthipeptide. The reaction was quenched by the addition of 3-mercaptopropionic acid. (F) MS^2^ spectra with the key fragment ions annotated that demonstrate C–C bond formation to be affected by the Trp side chain indole.

The bromine atom could be added by MppI to either of two indole side chains in the MppE cores, namely, Trp80 and Trp82. To determine which of these two Trp residues was brominated, Trp80 and Trp82 were individually mutated to Phe, and the genes encoding the mutant MppE precursor peptides were coexpressed with *mppM* and *mppI* (Table S1). The Trp80 → Phe mutation greatly reduced the production of a brominated product, while robust brominated product formation was observed for the Trp82 → Phe mutant (Figure S8). These observations allowed us to posit that bromination is affected by the Trp80 side chain (Figure 2). Enzymatic degradation of the brominated product followed by a retention time comparison with authentic synthetic standards demonstrated regiospecific indole-5 bromination (Figure S9). Taken together, these data led to the structural characterization of the brominated lanthipeptide furnished by MppM and MppI (Figure 2).

To exploit the utility of the aryl bromide, we screened for reaction conditions for palladium-assisted Suzuki–Miyaura cross coupling (SMCC) of 4-methoxyphenyl boronic acid to the brominated lanthipeptide furnished by MppM and MppI. As peptides containing tryptophan residues are prone to oxidative degradation, as we had observed previously, we were mindful of the reaction conditions not to necessitate high temperatures (Table S2).^6^ Various Pd ligands were evaluated. Of these, the previously described 2-dimethylamino-4,6-dihydroxypyrimidine demonstrated the requisite product formation (Figure 2E).^19,20^ Product identity was confirmed by high-resolution mass spectrometry; fragmentation of the [M + H]^1+^ parent ion led to detection of characteristic immonium product ions corresponding to the unmodified Trp82 side chain indole as well as the Trp80 side chain indole, which now bears the methoxyphenyl adduct (Figure 2F). Note that immonium ions for the Arg side chain are usually not observed in this experiment, and the immonium ions for Ala and Gly residues are too small to be detected.^21^ Elimination of the catalyst from the reaction abolished the formation of the methoxyphenyl adduct (Figure S10).

Along with the macrocyclic brominated lanthipeptide, coexpression of *mppE* with *mppM* and *mppI* led to the detection of a linear brominated product as well; here, bromination proceeds without macrocyclization (Figure S11). Hence, the order of MppM- and MppI-catalyzed transformations and the substrate preference of MppI (macrocyclic or linear MppE) was unclear. The recombinant MppI enzyme was purified, and bromination activity was reconstituted *in vitro* for the linear MppE and macrocyclic MppE substrates (Figure 3A). The *in vitro* assays involved the flavin reductase RebF and the NADH-generating phosphite dehydrogenase PTDH as reaction partners.^22,23^ In a competition experiment, wherein equimolar linear MppE and macrocyclic MppE were provided as substrates to MppI, we detected the preferential bromination of the macrocyclized MppE substrate (Figure 3B, Figures S12 and S13). Furthermore, when substrate conversions were evaluated for these two substrates in separate individual *in vitro* bromination reactions, the conversion for the macrocyclized MppE substrate was greater (Figure 3C, Figures S14 and S15). These data allow us to posit that the physiological substrate for MppI is the macrocyclized MppE peptide and that macrocyclization precedes bromination.

**Figure 3:**
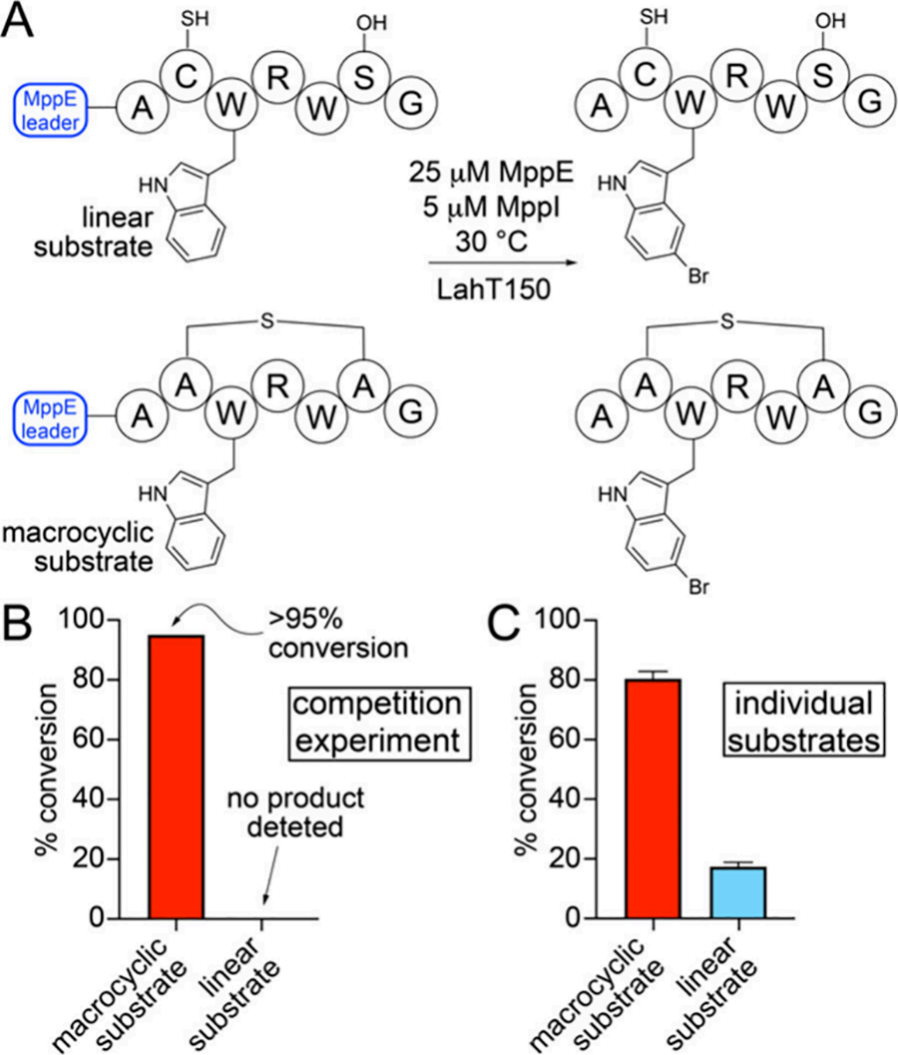
(A) Scheme for the bromination of linear and macrocyclic MppE substrates by MppI, followed by leader peptide excision by LahT150. (B) Macrocyclic and linear substrate conversion when these substrates were provided to MppI in an equimolar amount in a competition experiment. All biochemical experiments were performed in triplicate, and mean and standard deviations from independent measurements are plotted. (C) Conversions when macrocyclic and linear substrates were provided in separate experiments to MppI.

We next evaluated the leader peptide dependence for MppI. RiPP precursor peptides, such as MppE, are divided into a C-terminal core sequence that is post-translationally modified and an N-terminal leader sequence that is not modified but serves to facilitate peptide–enzyme interaction. When the macrocyclized MppE core alone without the MppE leader was provided as a substrate to MppI, no brominated product formation was observed (Figure S16). This observation implies that MppI binds to the MppE leader, which is in contrast to the activity of the flavin-dependent halogenase MibH that chlorinates a macrocyclized lanthipeptide only after the leader is proteolytically removed from the modified core peptide.^24^

The precursor peptides MppE and SrpE both possess long leaders (SrpE is the precursor peptide for SrpI).^8^ Much of the long SrpE leader peptide was dispensable for binding to SrpI as long as the two Leu residues comprising of the L(X)_4_L motif were preserved (Figure 4A).^25^ While SrpE belongs to the nitrile hydratase leader peptide (NHLP) family and MppE belongs to the Nif11-like peptide family, the L(X)_4_L motif is conserved in the MppE leader as well.^13^ Mutation of the two Leu residues comprised of the L(X)_4_L motif in MppE (Leu66 and Leu71) to Ala only had a modest deleterious effect on MppI activity (Figure 4B, Table S1, Figure S17); the corresponding L(X)_4_L → A(X)_4_A mutation in SrpE had abolished SrpI activity.^25^

**Figure 4:**
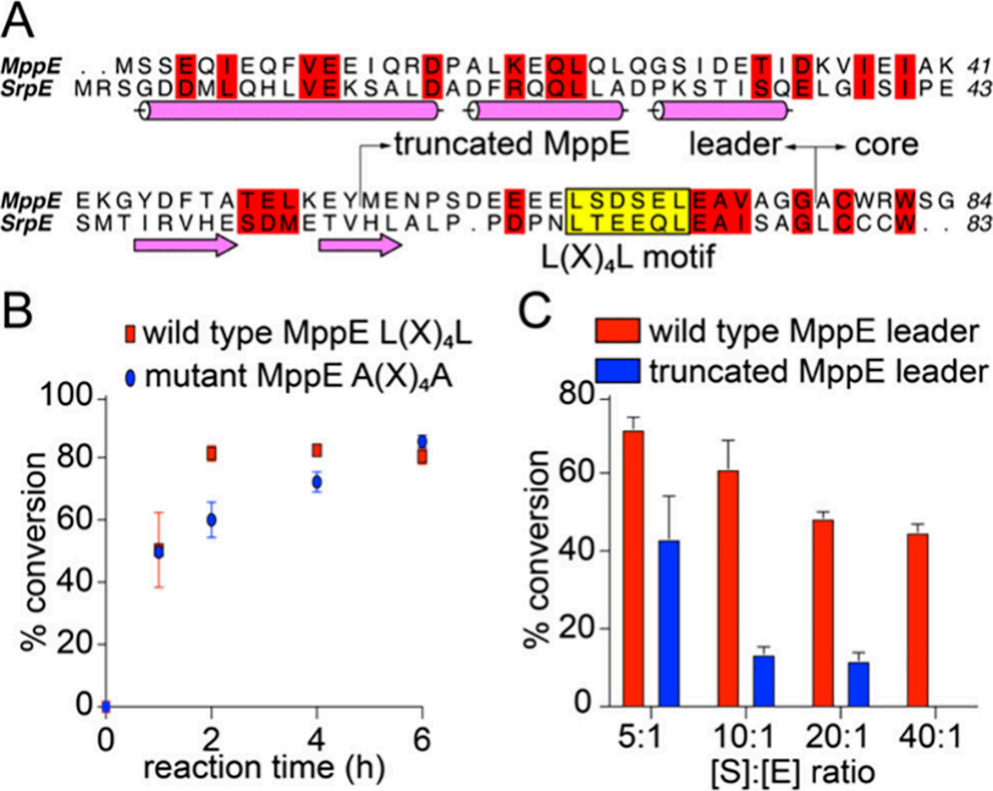
(A) Sequence alignment between MppE and SrpE peptides. The secondary structural elements determined for the SrpE leader are annotated. The conservation of the L(X)_4_L motif is highlighted. (B) Time-dependent halogenation of the macrocyclic MppE substrate by MppI bearing the wild-type MppE leader and the when the L(X)_4_L motif in MppE leader was mutated to A(X)_4_A. All biochemical experiments were performed in triplicate, and mean and standard deviations from three independent measurements are plotted. (C) Truncation of the MppE leader (site of truncation denoted in panel A) compromises MppI activity at high [S]/[E] stoichiometric ratios.

Next, guided by the sequence alignment between SrpE and MppE, the MppE leader peptide was truncated (Table S1); a similarly truncated SrpE leader that preserved the L(X)_4_L motif was a competent substrate for SrpI.^25^ Evaluating the truncated MppE substrate peptide at different substrate-to-enzyme stoichiometric ratios revealed that leader truncation led to a loss of MppI activity (Figure 4C). Thus, while MppI and SrpI are both leader-dependent, there is dissonance in the mode of leader engagement between the two enzymes. These biochemical observations are in alignment with AlphaFold3-predicted models for MppI/MppE-leader and SrpI/SrpE-leader complexes wherein MppI contacts the entire MppE leader sequence, while SrpI only contacts a small C-terminal section of the SrpE leader peptide (Figure S18).

While the potential of MppI to furnish diverse brominated macrocyclic peptides remains to be evaluated, findings from this study support the assertion that transformation-guided genome mining can lead to the discovery of new biocatalytic modalities. The Trp indole is the site for numerous enzymatic transformations that are critical for the bioactivities of the corresponding natural products.^26^ However, unlike bioorthogonal manipulation of Cys and Lys side chains that offer a singular reactive site, regiospecific transformation of indole side chains in peptides and proteins is hard to reach.^27^ While strategies to modify the indole-2 position have been developed, at the moment, regiospecific access to the indole-4–7 positions seems be largely restricted to enzymes.^28−31^ With the goal of developing bioorthogonal chemistries that can interface with peptides and proteins, RiPP biosynthetic enzymes are a viable starting point.

## Data Availability

The data underlying this study are available in the published article and its Supporting Information.

## Supporting Information Available

Comprehensive description of materials and methods used in this study, synthetic schemes, compound characterization data, and descriptions of enzyme reaction outcomes (PDF)
