# Supplementary material for: Transformation-Guided Genome Mining Provides Access to Brominated Lanthipeptides
Source: Org Lett. 2025 Jan 17;27(4):984–8. doi: 10.1021/acs.orglett.4c04529 (PMC11791876; doi:10.1021/acs.orglett.4c04529)
Supplement: Supplementary file 1 — ol4c04529_si_001.pdf [file ol4c04529_si_001.pdf]

SUPPLEMENTARY INFORMATION FOR:

Transformation-guided genome mining provides access to brominated lanthipeptides

Nirmal Saha,[1,†] FNU Vidya,[1,†] Youran Luo,[2] Wilfred A. van der Donk,[2,3,4] Vinayak Agarwal[1,5,*]

[1] School of Chemistry and Biochemistry, Georgia Institute of Technology, Atlanta, Georgia 30332, United States

[2] Department of Chemistry, University of Illinois at Urbana–Champaign, Urbana, Illinois 61801, United States

[3] Carl R. Woese Institute for Genomic Biology, University of Illinois at Urbana–Champaign, Urbana, Illinois 61801, United States

[4] Howard Hughes Medical Institute, University of Illinois at Urbana–Champaign, Urbana, Illinois 61801, United States

[5] School of Biological Sciences, Georgia Institute of Technology, Atlanta, Georgia 30332, United States

[†] Equal contribution authors

* correspondence: vagarwal@gatech.edu, Ph: 404-385-3798

TABLE OF CONTENTS

Supplementary Materials and Methods S2

Supplementary Tables S9

Supplementary Figures S11

Supplementary References S27

SUPPLEMENTARY MATERIALS AND METHODS

General materials and instrumentation

All chemicals, solvents, and media components were obtained from Sigma-Aldrich, Fisher Scientific, or VWR, and used without further purification. Phusion high-fidelity DNA polymerase and Gibson assembly Master Mix were procured from New England Biolabs. PrimeSTAR DNA polymerase Master Mix was procured from Takara Bio. Synthetic DNA fragments were procured from Twist Biosciences. Mass spectra were recorded on an Agilent 6530C time of flight (ToF) mass spectrometer equipped with an electrospray ionization (ESI) source coupled to an Agilent 1260 liquid chromatography system equipped with a diode array detector (DAD) unless specified.

Genome mining to identify biosynthetic gene clusters (BGCs) harboring genes encoding halogenases and peptide macrocyclases

The SrpI sequence, described previously, was used the query sequence to mine for similar sequences using the Enzyme Function Initiative-Enzyme Similarity Tool (EFI-EST) from the UniProt database with the e-value for UniProt sequence retrieval set to 20 and the e-value for sequence similarity edge calculation set to 5.[1] Using this strategy, 123 sequences were retrieved of which 119 were unique sequences. These sequences were organized into a sequence similarity network (SSN) with an alignment score of 75. The SSN was visualized using Cytoscape and is illustrated in Figure 1A.[2] Gene neighborhood diagrams (GNDs) were generated using default parameters and the biosynthetic gene clusters (BGCs) encoding SrpI-like halogenases were manually curated.

Plasmid DNA construction

Synthetic DNA fragments encoding *mppE*. *mppM*, and *mppI* optimized for expression in *Escherichia coli* were obtained from commercial vendors. Standard molecular biology techniques were used for cloning and plasmid construction. Amplification of target DNA fragments was carried out using either Phusion or PrimeSTAR high-fidelity DNA polymerases. Gibson assembly was used to subclone genes encoding proteins of interest into the target vectors. The sequences of recombinant plasmids were confirmed either by Sanger sequencing or by Nanopore sequencing. Wild type and mutant forms of the precursor peptide MppE encoding genes were cloned in pET28(+) vector. For *in vivo* co-expression expression, *mppM* was cloned into the multiple cloning site-2 (MCS-2) of the pCDF-Duet vector with or without *mppI* cloned into the MCS-1. For expression and recombinant protein purification, *mppI* was also

cloned into the pET28(+) vector. Gene encoding the MppE-truncated peptide was cloned in the pET28-MBP vector.

Expression and purification of brominase MppI, reaction partners RebF and PTDH, and peptidase LahT150

For overexpression of N-His_6-MppI, the pET28(+)-*mppI* vector was co-transformed with plasmid pGro7 (Takara Biosciences) into *E. coli* BL21(DE3). Colonies were grown under antibiotic kanamycin (50 μg/mL, final concentration) and chloramphenicol (34 μg/mL, final concentration) on Luria Bertani (LB) agar media for 16 h at 37 °C. A single colony was picked and inoculated in 10 mL of terrific broth (TB) media supplemented with kanamycin and chloramphenicol for 16 h at 37 °C. This inoculum was used to initiate 1 L TB media supplemented with kanamycin and chloramphenicol. Cultures were incubated with shaking at 30 °C until optical density measured at 600 nm (OD_{600}) reached 0.6. Cultures were cooled at 18 °C for 1 h before protein expression was inducted by addition of 0.3 mM (final concentration) isopropyl-β-*d*-1-thiogalactopyranoside (IPTG) and 250 mg L-arabinose. The culture was further incubated for 48 h at 18 °C with shaking.

For protein purification, *E. coli* cultures were harvested by centrifugation and cell pellets were resuspended in lysis buffer A (20 mM Tris-Cl (pH 7.9), 500 mM NaCl). Cells were lysed by homogenization. The lysate was clarified by centrifugation at 25,000×g for 60 min at 4 °C. The supernatant was loaded onto a 5 mL His-Trap Ni-NTA column at 4 °C. The column was washed extensively with wash buffer B (20 mM Tris-Cl (pH 7.9), 500 mM NaCl, 30 mM imidazole), and protein was eluted using a linear gradient from 0% to 100% elution buffer B (20 mM Tris-Cl (pH 7.9), 500 mM NaCl, 250 mM imidazole). Protein purity was analyzed by denaturing gel electrophoresis and fractions containing protein of interest were pooled and dialyzed overnight in the buffer containing 20 mM Tris-Cl (pH 8.9), 50 mM KCl.

After overnight dialysis, the protein was loaded onto a 5 mL Hi-Trap IEX column equilibrated with the binding buffer (20 mM Tris-Cl (pH 8.9), 50 mM KCl) at 4 °C. After extensive washing with binding buffer, bound proteins were eluted using elution buffer (20 mM Tris-Cl (pH 8.9), 1 M KCl) in four fractions of 6 mL volume each. The purity of eluent fractions was checked by denaturing gel electrophoresis and pure fractions pooled. Protein concentration was measured by Bradford assay. Flavin reductase RebF, phosphite dehydrogenase PTDH, and peptidase LahT150 were purified as described previously.[3]

Procedure for expression and purification of linear and macrocyclic MppE peptides

To obtain linear MppE peptides, pET28(+)-*mppE* (wild type and mutants thereof) plasmids were co-transformed into *E. coli* BL21(DE3) together with empty pCDF-Duet plasmid. To obtain macrocyclized MppE peptides, pET28(+)-*mppE* plasmids (wild type and mutants thereof) were co-transformed into *E. coli* BL21(DE3) together with pCDF-Duet-*mppM* plasmid. To obtain macrocyclized MppE peptide with truncated leader peptide, pET28-MBP-*mppE*$^{(truncated)}$ plasmid was co-transformed into *E. coli* BL21DE3 along with pCDF-Duet-*mppM* plasmid. Bromination of truncated MppE substrate was compared against MBP-fused full length MppE substrate. To obtain macrocyclized and brominated MppE peptides, pET28(+)-*mppE* plasmid was co-transformed into *E. coli* BL21DE3 along with empty pCDF-Duet-*mppM*+*mppI* plasmid; plasmid construction has been described previously. All *E. coli* strains were further transformed with the pGro7 chaperone plasmid. Mutant forms of the MppE peptide refer to the W80F, W82F, and $L(X)_4L \rightarrow A(X)_4A$ variants used in this study and the peptide sequences along with the truncated MppE variant are listed in Supplementary Table 1.

Colonies were grown under appropriate antibiotic selection on LB agar media for 16 h at 37 °C. A single colony was picked and inoculated in 10 mL of TB supplemented with appropriate antibiotics for 16 h at 37 °C. This inoculum was used to initiate 2 L TB cultures supplemented with corresponding antibiotics. When *mppI* was designed to be expressed, the *E. coli* growth media was supplemented with 1 g/L KBr and 10 mg/L riboflavin. Cultures were incubated with shaking at 37 °C until the OD_{600} reached 0.4 when 250 mg/L L-arabinose was added. Cultures were further incubated at 37 °C until OD_{600} reached 0.6. Cultures were cooled at 18 °C for 1 h before protein expression induction by addition of 0.5 mM IPTG. Cultures were incubated at 18 °C, 180 rpm for 16 h before harvesting by centrifugation 4,000×g for 30 min at 4 °C.

Protein purification procedures were performed at 4 ˚C or on ice. Cell pellets were resuspended in binding buffer (20 mM Tris-Cl (pH 7.5), 1 M NaCl) and lysed by sonication. The lysate was clarified by centrifugation at 25,000×g for 45 min, and the supernatant was loaded to a 5 mL HisTrap Ni-NTA column equilibrated in lysis buffer. The column was washed extensively with wash buffer (20 mM Tris-Cl (pH 7.5), 30 mM imidazole, 1 M NaCl) and then eluted with a linear gradient to 100% elution buffer (20 mM Tris-Cl (pH 7.5), 500 mM imidazole, 1 M NaCl). Protein purity was analyzed by denaturing gel electrophoresis and fractions containing protein of interest were pooled and dialyzed overnight against binding buffer before storage.

Proteolytic excision of the leader peptide and LC/MS analysis

LahT150-catalyzed cleavage reactions were performed in 200 µL volume reactions containing 50 mM HEPES-Na (pH 7.5), 5 mM DTT, 26 µM of LahT150 protease and 169 µM MppE peptide(s). The

MppE $L(X)_4L \rightarrow A(X)_4A$ peptide was digested with Glu-C as described previously.[3] Reactions were incubated at 30 °C for 3 h in a dry bath. An equal volume of MeOH + 1% (v/v) TFA was added to quench the reactions, and precipitates were removed by centrifugation at 16,000×g for 30 min at room temperature. The supernatants were withdrawn to high-performance liquid chromatography (HPLC) vials for analysis by liquid chromatography/mass spectrometry (LC/MS).

Mass spectrometry data were collected in the positive ionization mode in the MS^1 mass range *m/z* 50–3000 Da and MS^2 mass range *m/z* 50–3000. Chromatography was performed using Agilent Poroshell 120 2.7 µm C_{18} reversed-phase column (100 × 4.6 mm) at a flow rate of 0.3 mL/min using the following solvents for the mobile phase: solvent A: H_2O + 0.1% (v/v) formic acid; solvent B: MeCN + 0.1% (v/v) formic acid. The chromatography elution profile was as follows: 5% solvent B from 0 to 5 min, linear gradient to 100% solvent B from 5 to 18 min, 100% solvent B from 18 to 22 min, linear gradient to 5% solvent B from 22 to 24 min, and 5% solvent B from 24 to 30 min. Mass spectrometry data were acquired from between 5 min and 24 min. For MppE peptides, the $[M+2H]^{2+}$ parent ions dominated the MS^1 spectra. The area under the extracted ion chromatograms (EICs) for the $[M+2H]^{2+}$ MS^1 ions were used to quantify the relative amounts of the substrate and product peptides.

Preparative scale isolation of the modified MppE core peptides (cyclic-ACWRWSG, and cyclic and brominated-ACWRWSG)

Modified MppE peptides were purified from *E. coli* cultures as described above, digested with LahT150, and the digestion reaction lyophilized. To recover the core peptides, the mixture was separated by semi-preparative HPLC and the fractions analyzed by LC/MS for the presence of the requisite core peptide(s). HPLC separations were carried out using Phenomenex Luna 5 µm C_{18} 100 Å LC column (250 × 10 mm) using Agilent 1260 Infinity II HPLC system. Water (solvent A) and MeCN (solvent B) supplemented with 0.1% (v/v) trifluoroacetic acid (TFA) were used as mobile phases. A flow rate of 2 mL/min was used throughout with the following gradient: 0–10 min: 5% B, 10–20 min: 15% B, 20–30 min: 22% B, 30–40 min: 30% B, 40–50 min: 35 % B, 50–55 min: 40% B, 55–60 min: linear gradient to 100% B, followed by 10 min each of column washing with 100% B and equilibration. UV absorbance was monitored at 210 and 254 nm.

Lanthionine stereochemistry determination

The lanthionine configuration of the cyclic-ACWRWSG MppE core peptide, purified as above, was determined using methods described previously.[4] Briefly, the peptide was hydrolyzed using 6 M DCl

at 120 °C for 20 h and derivatized with 1-fluoro-2-4-dinitrophenyl-5-L-leucine amide (L-FDLA) for 3 h. The derivatized sample was then injected into Agilent 6545 LC/Q-TOF instrument with the Kinetex F5 Core−Shell HPLC column (1.7 µm F5 100 Å, LC Column 100 mm×2.1 mm) for the LC/MS analysis. mCylLs (plasmid available at Addgene ID #208760) and mCoiA1 (Addgene ID #208761 and #208762) were prepared as the lanthionine and methyllanthionine standards, respectively, for comparison and co-injection.

MppI-catalyzed bromination of linear and macrocyclic MppE peptides

MppI-catalyzed bromination reactions were performed in 200 µL volume containing 50 mM HEPES-Na (pH 7.9), 20 mM KBr, 25 µM FAD, 0.5 mM NAD^+, 5 mM Na_2HPO_3, 2.5 µM RebF, 2.5 µM PTDH, 25 µM peptidic substrates, 5 µM MppI, and 0.01 µg/µL catalase. After 12 h incubation at 30 °C in a dry bath, reactions were quenched by adding the protease LahT150 with DTT (1 µL, 1 M). After 4 h incubation at room temperature, the reactions treated with LahT150 were added an equal volume of MeCN + 2% (v/v) TFA to the reaction tube. The precipitates were removed by centrifugation at 16,000×g for 30 min. The supernatants were withdrawn to HPLC vials. For negative control reactions, MppI was omitted. For time course assays, aliquots were withdrawn at designated time points and analyzed analogously. Bromination assays for the cyclized MppE core without the leader peptide was conducted in an identical manner. For assays with the $MppE^{(truncated)}$ peptide, the MBP-labeled substrate was employed to enhance the solubility of the truncated peptide.

Competition experiment to compare MppI-catalyzed bromination of linear and macrocyclic MppE peptides

MppI-catalyzed bromination reactions were performed in 200 µL volume containing 50 mM HEPES-Na (pH 7.9), 20 mM KBr, 25 µM FAD, 0.5 mM NAD^+, 5 mM Na_2HPO_3, 2.5 µM RebF, 2.5 µM PTDH, 25 µM peptidic substrates, 5 µM MppI, and 0.01 µg/µL catalase. In this experiment, the 25 µM peptidic substrate refers to an equimolar mixture (12.5 µM each) of linear MppE and macrocyclic MppE peptides. Protease LahT150 was added after 6 h incubation at 30 °C in a dry bath with DTT (1 µL, 1 M). After 2 h incubation at room temperature, the reactions treated with LahT150 were quenched by adding an equal volume of MeOH + 2% (v/v) TFA in the reaction tube. The precipitates were removed by centrifugation at 16,000×g for 30 min. The supernatants were withdrawn to HPLC vials and analyzed analogously. All biochemical experiments were performed in triplicate and mean and standard deviations from independent measurements were plotted.

MppI-catalyzed bromination of truncated MppE peptides

Wild type and truncated MppE peptides were cloned into modified pET28 vectors that afford an N-terminal N-His_6-maltose binding protein (N-His_6-MBP) fusion tag (Table S1). The N-His_6-MBP fusion improves the solubility of the truncated MppE peptide. The genes encoding the N-His_6-MBP tagged peptides were coexpressed with *mppM* and the peptides purified as before. MppI-catalyzed bromination reactions were performed in 200 µL volume containing 50 mM HEPES-Na (pH 7.9), 20 mM KBr, 25 µM FAD, 0.5 mM NAD^+, 5 mM Na_2HPO_3, 2.5 µM RebF, 2.5 µM PTDH, 5 µM MppI, and 0.01 µg/µL catalase with 25 µM, 50 µM, 100 µM, 200 µM peptidic substrates. LahT150 was added after 6 h incubation at 30 °C in a dry bath with DTT (1 µL, 1 M). After 2 h incubation at room temperature, the reactions treated with LahT150 were quenched by adding an equal volume of MeOH + 2% (v/v) TFA in the reaction tube. The precipitates were removed by centrifugation at 16,000×g for 30 min. The supernatants were withdrawn to HPLC vials and analyzed analogously. For wild-type and truncated MppE peptides, conversion was monitored when these peptide substrates were provided to MppI in a (1:5, 1:10, 1:20, 1:40) ratio. All biochemical experiments were performed in triplicate and mean and standard deviations from independent measurements were plotted.

Synthesis of 2-, 4-, 5-,6-, and 7-bromo-L-tryptophan

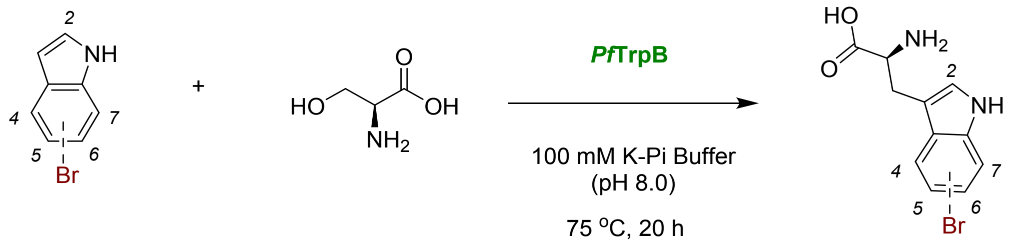

Standards of 4-, 5-, 6-, and 7-bromo-L-tryptophan were prepared enzymatically using the tryptophan synthase *Pf*TrpB.[5] The reaction mixture consisted of 1 mM of either 4-, 5-, 6-, or 7-bromoindole (commercial, Sigma-Aldrich) and 1 mM L-serine in 100 mM potassium phosphate buffer at pH 8.0. The reaction was initiated by addition of 5 µM purified *Pf*TrpB; expression and purification of *Pf*TrpB has been described previously.[6] The reaction mixture was incubated at 75 °C for 20 h. Following incubation, the reaction was quenched by adding an equal volume of a 1:1 mixture of 1 M HCl and MeOH. The mixture was centrifuged to remove precipitates and used directly for LC/MS.

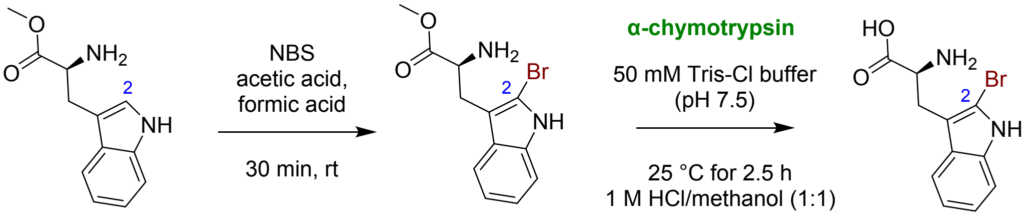

The synthesis of 2-bromo-L-tryptophan proceeded as follows: in a 10 mL round-bottom flask, 130 mg of L-tryptophan methyl ester (0.51 mmol) was dissolved in 5 mL of glacial acetic acid and 1.5 mL of formic acid. To this solution was added 92.5 mg of N-bromo succinimide (0.52 mmol) with stirring. The reaction was allowed to proceed at room temperature for 30 min. After this time, the solvents were removed under reduced pressure. The resulting product was purified by isocratic silica column chromatography using a 95:5 DCM:MeOH to obtain 2-bromotryptophan methyl ester. The ester was then hydrolyzed using α-chymotrypsin. The cleavage reaction contained 2 mM of 2-bromo-L-tryptophan methyl ester and 0.5 mg/mL of chymotrypsin in 50 mM Tris-Cl (pH 7.5). The reaction was incubated at 25 °C for 2.5 h and subsequently quenched by adding an equal volume of a 1:1 mixture of 1 M HCl and MeOH. The mixture was then centrifuged to remove any precipitates, and used directly for LC/MS.

Carboxypeptidase A catalyzed digestion of cyclized ACW(Br)RWSG [(core)]

The macrocyclized and brominated MppE core peptide, cyclic-ACW$_{(Br)}$RWSG at a concentration of 50 µM was incubated with 100 ng of carboxypeptidase A in 50 mM Na-acetate (pH 5.5) buffer for 16 h at 30 °C in a dry bath. The digestion reaction was quenched by addition of equal volume of a 1:1 mixture of 1 M HCl and MeOH. The mixture was centrifuged to remove any precipitates, and used directly for LC/MS. The retention time of the carboxypeptidase A-excised BrTrp was compared to the BrTrp standards described above for determination of MppI-catalyzed bromination regiospecificity.

Suzuki-Miyaura cross-coupling reactions

Several water-soluble phosphine-based (**L1–L4**) and nitrogen-based (**L5**, **L6**) ligands have been developed to facilitate Suzuki-Miyaura cross-coupling reactions. These ligands were obtained commercially.

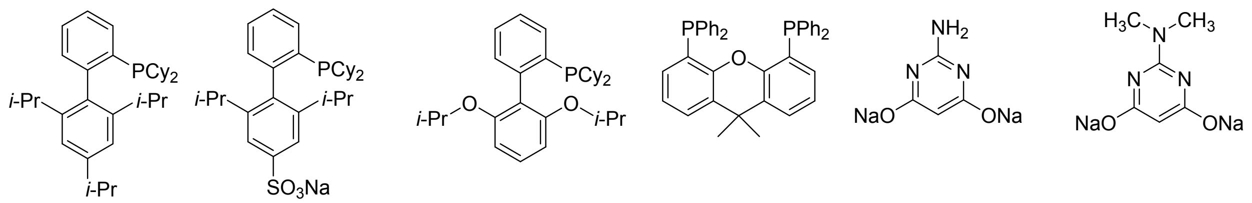

L1 (XPhos) **L2** (sXPhos) **L3** (RuPhos) **L4** (XantPhos) **L5** (ADHP) **L6** (DMADHP)

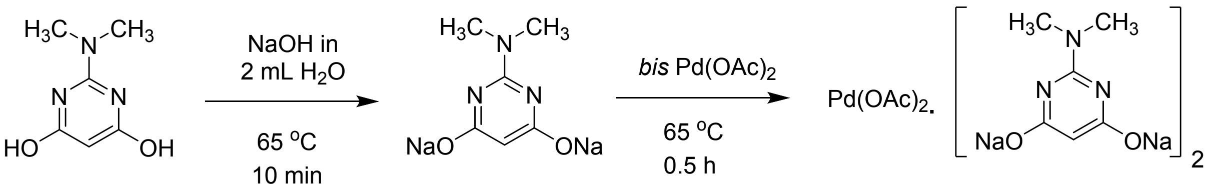

2-(dimethylamino)pyrimidine-4,6-diol (15.5 mg, 0.1 mmol) was dissolved in NaOH (2 mL, 0.1 M) at 65 ºC. Palladium acetate (11 mg, 0.05 mmol) was added, and the solution was stirred at 65 ºC for 30 min in a water bath. The orange solution was then cooled and diluted to 5 mL with distilled water to give a stock 10 mM catalyst solution (**L6**). **L5** was synthesized following the same procedure.

Procedure for Suzuki coupling of aryl boronic acid with brominated cyclic peptide

The cross-coupling reactions were performed in 200 µL volume containing 50 µM cyclic-ACW(Br)RWSG MppE core peptide, 0.5 mM Pd-**L6** (0.1 µmol, 10 µL), K_2CO_3 (0.3 mM, 6 eq.), and 0.5 mM 4-methoxyphenyl boronic acid. The reaction was incubated at 45 °C for 6 h. 10 µL of 3-mercaptopropionic acid (10 µL/mL) was added to chelate the excess Pd. The reaction mixture was centrifuged for 30 min, and the aliquots were analyzed by LC/MS. Areas under the EICs corresponding to the unmodified substrate and the brominated product were used to calculate the yield of the coupling reaction. Under optimized conditions, the ligands **L1**–**L5** did not demonstrate product formation.

Supplementary Tables

Supplementary Table S1: Precursor peptide sequences used in this study with core sequences in bold

	**Amino acid sequence**
N-His$_6$-MppE	MGSSHHHHHH†SSGLVPRGS§HMMSSEQIEQFVEEIQRDPALKEQLQL<br>QGSIDETIDKVIEIAKEKGYDFTATELKEYMENPSDEEEELSDSELEAV<br>AGG**ACWRWSG**
N-His$_6$-MppE<br>W80F mutant	MGSSHHHHHH†SSGLVPRGS§HMMSSEQIEQFVEEIQRDPALKEQLQL<br>QGSIDETIDKVIEIAKEKGYDFTATELKEYMENPSDEEEELSDSELEAV<br>AGG**AC*F*RWSG**
N-His$_6$-MppE<br>W82F mutant	MGSSHHHHHH†SSGLVPRGS§HMMSSEQIEQFVEEIQRDPALKEQLQL<br>QGSIDETIDKVIEIAKEKGYDFTATELKEYMENPSDEEEELSDSELEAV<br>AGG**ACWR*F*SG**
N-His6-MppE<br>L(X)$_4$L→A(X)$_4$A mutant	MGSSHHHHHH†SSGLVPRGS§HMMSSEQIEQFVEEIQRDPALKEQLQL<br>QGSIDETIDKVIEIAKEKGYDFTATELKEYMENPSDEEEE*A*SDSE*A*EAV<br>AGG**ACWRWSG**
N-His$_6$-MBP-MppE	MGSSHHHHHH†SSGLVPRGS§HMKIEEGKLVIWINGDKGYNGLAEVG<br>KKFEKDTGIKVTVEHPDKLEEKFPQVAATGDGPDIIFWAHDRFGGYA<br>QSGLLAEITPDKAFQDKLYPFTWDAVRYNGKLIAYPIAVEALSLIYNK<br>DLLPNPPKTWEEIPALDKELKAKGKSALMFNLQEPYFTWPLIAADGG<br>YAFKYENGKYDIKDVGVDNAGAKAGLTFLVDLIKNKHMNADTDYSI<br>AEAAFNKGETAMTINGPWAWSNIDTSKVNYGVTVLPTFKGQPSKPF<br>VGVLSAGINAASPNKELAKEFLENYLLTDEGLEAVNKDKPLGAVAL<br>KSYEEELAKDPRIAATMENAQKGEIMPNIPQMSAFWYAVRTAVINA<br>ASGRQTVDEALKDAQTNSSSHHHHHH†ANSVPLVPRGS§ENLYFQS‡G<br>SMMSSEQIEQFVEEIQRDPALKEQLQLQGSIDETIDKVIEIAKEKGYDF<br>TATELKEYMENPSDEEEELSDSELEAVAGG**ACWRWSG**
N-His$_6$-MBP-MppE(truncated)	MGSSHHHHHH†SSGLVPRGS§HMKIEEGKLVIWINGDKGYNGLAEVG<br>KKFEKDTGIKVTVEHPDKLEEKFPQVAATGDGPDIIFWAHDRFGGYA<br>QSGLLAEITPDKAFQDKLYPFTWDAVRYNGKLIAYPIAVEALSLIYNK<br>DLLPNPPKTWEEIPALDKELKAKGKSALMFNLQEPYFTWPLIAADGG<br>YAFKYENGKYDIKDVGVDNAGAKAGLTFLVDLIKNKHMNADTDYSI<br>AEAAFNKGETAMTINGPWAWSNIDTSKVNYGVTVLPTFKGQPSKPF<br>VGVLSAGINAASPNKELAKEFLENYLLTDEGLEAVNKDKPLGAVAL<br>KSYEEELAKDPRIAATMENAQKGEIMPNIPQMSAFWYAVRTAVINA<br>ASGRQTVDEALKDAQTNSSSHHHHHH†ANSVPLVPRGS§ENLYFQS‡G<br>SMENPSDEEEELSDSELEAVAGG**ACWRWSG**

†His$_6$ tag, §thrombin cleavage site, ‡TEV cleavage site

Supplementary Table S2: Conditions[a] for the Suzuki cross-coupling reaction of cyclic-ACW(Br)RWSG peptide substrate with 4-methoxyphenyl boronic acid

Entry	Substrate	Cat-L6(eq.)	Base	4-MPBA (eq.)	Temp.	Time/h	Conv./%
1	cyclic-ACW(Br)RWSG	2.5	K_2HPO_4	3	rt	6	N.D.
2	cyclic-ACW(Br)RWSG	2.5	K_2HPO_4	10	35	6	N.D.
3	cyclic-ACW(Br)RWSG	10	K_2HPO_4	10	35	6	N.D.
4	cyclic-ACW(Br)RWSG	10	K_2HPO_4	10	45	6	30[b]
5	cyclic-ACW(Br)RWSG	10	K_2CO_3	10	45	6	44.5[b]
6	cyclic-ACW(Br)RWSG	-	K_2CO_3	10	45	6	N.D.
7	cyclic-ACW(Br)RWSG	10	-	10	45	6	N.D.
8	cyclic-ACW(Br)RWSG	10	K_2CO_3	10	65	6	N.D.[c]

[a]substrate peptide (50 μM, 1.0 eq), 10.0 mM *bis*Pd(OAc)$_2$ catalyst-**L6** ligand solution (0.1 μmol, 10 μL), 4-methoxy phenylboronic acid (4-MPBA: 0.5 mM, 10 eq.,), K_2CO_3 (6.0 eq.), H_2O/CH_3CN (4:1, 0.2 mL), capped microcentrifuge tube, the temperature in ^{o}C, [b]yield determined by LC/MS, N.D. is not detected, N.D.[c] is not detected with substrate degradation.

SUPPLEMENTARY FIGURES

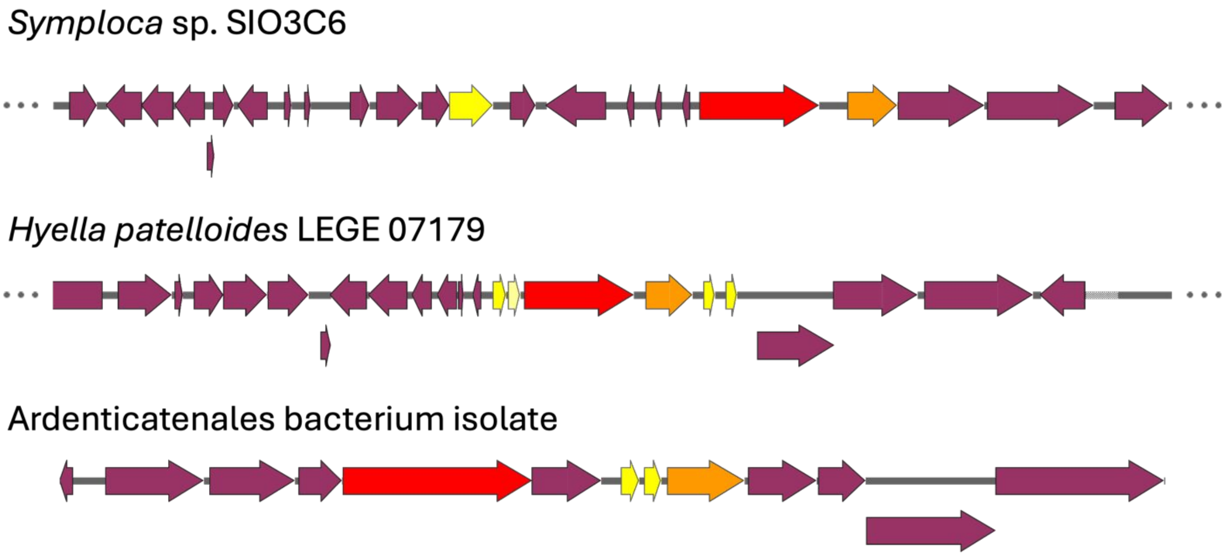

Figure S1: BGCs wherein genes encoding RiPP precursor peptides (in yellow) were detected to be clustered in neighborhood of genes encoding LanMs (in red) and flavin-dependent halogenases (in orange). The *mpp* BGC is illustrated in Figure 1B.

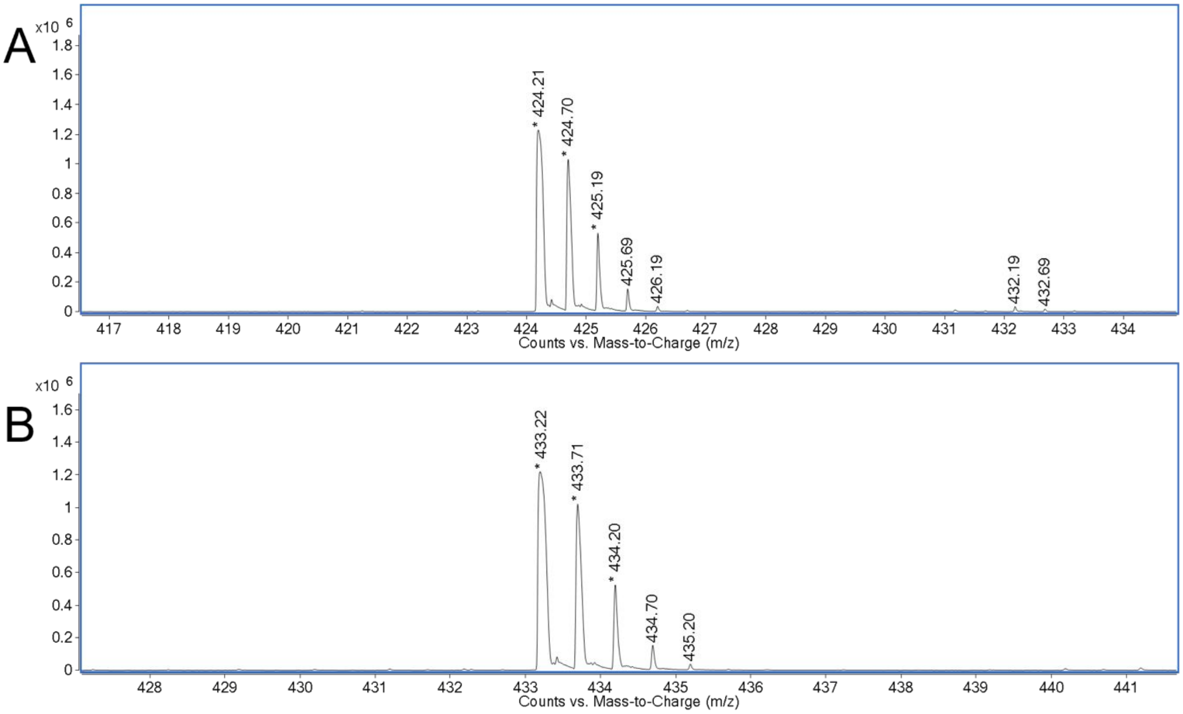

Figure S2: (**A**) MS^1 spectra demonstrating isotopic distribution of $[M+2H]^{2+}$ ions for MppM-modified macrocyclized MppE core peptide. (**B**) MS^1 spectra demonstrating isotopic distribution of $[M+2H]^{2+}$ ions for unmodified linear MppE core peptide. The MppE core peptides were excised from the full length MppE peptide using the peptidase LahT150.

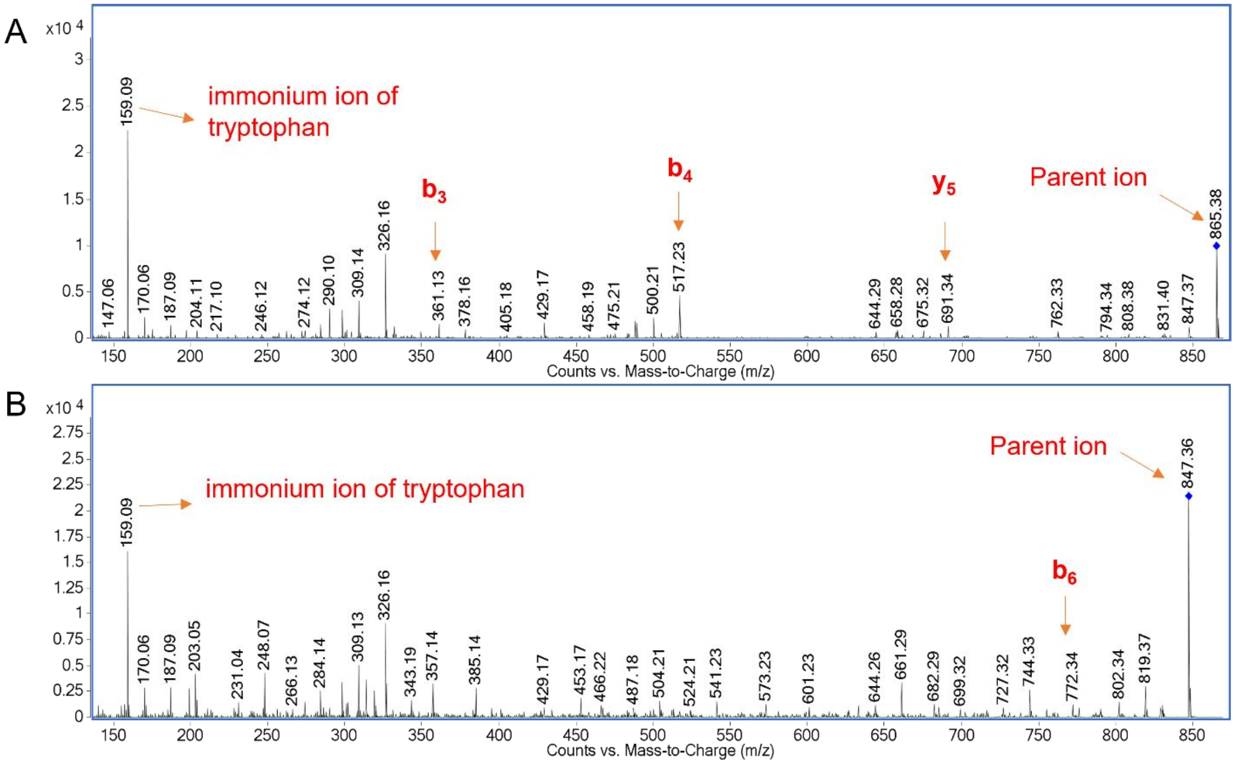

Figure S3: (**A**) MS[2] fragmentation spectrum for the $[M+H]^{1+}$ parent ion (labeled by blue diamond) corresponding to the unmodified linear MppE core peptide. The spectrum illustrates annotated product ions. (**B**) MS[2] fragmentation spectrum for the $[M+H]^{1+}$ parent ion (labeled by blue diamond) corresponding to the MppM-modified macrocyclized MppE core peptide. In both spectra, the immonium ion corresponding to the Trp residue was observed and is labeled.

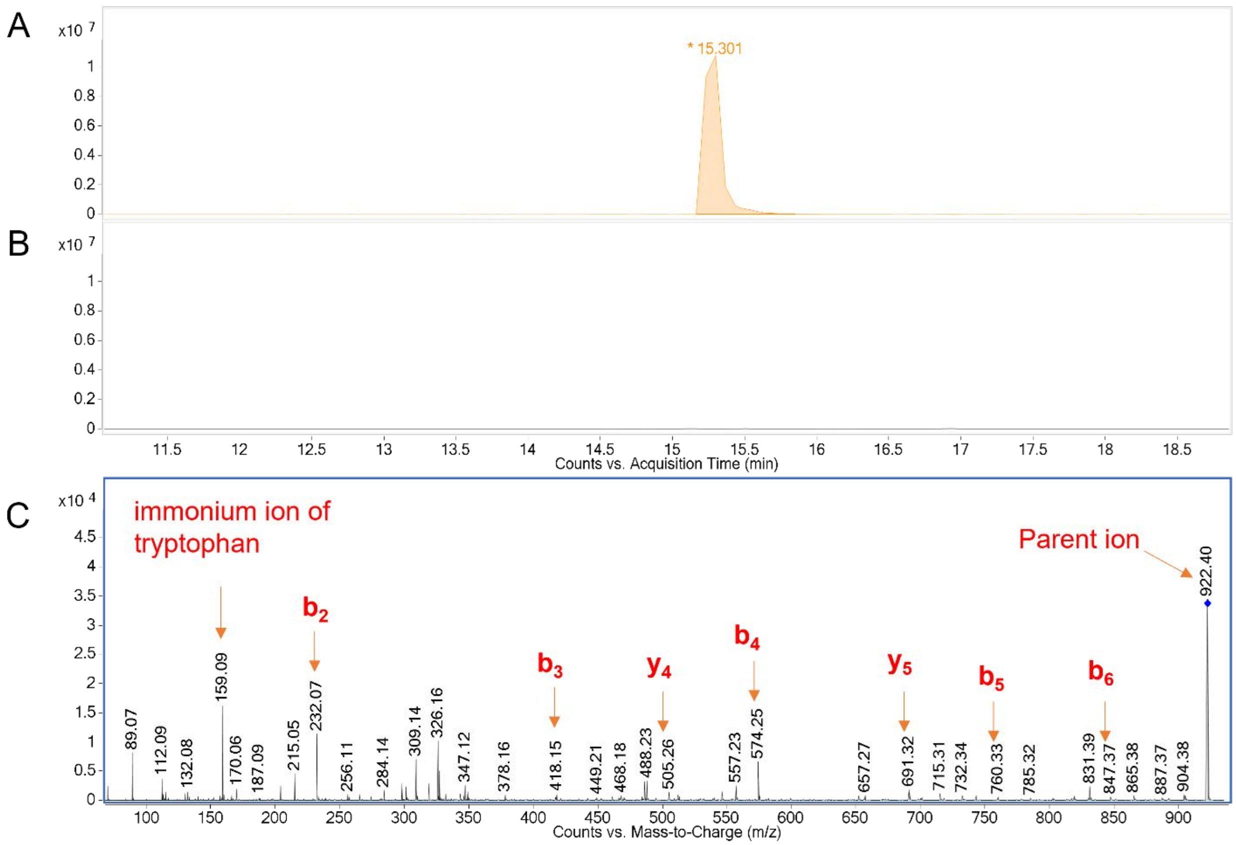

Figure S4: (**A**) Extracted ion chromatogram (EIC) for the $[M+2H]^{2+}$ parent ion corresponding to the unmodified MppE linear peptide wherein the Cys side chain bears an acetamide adduct after treatment with iodoacetamide (IAA). (**B**) EIC for the $[M+2H]^{2+}$ parent ion corresponding to the hypothetical dehydrated MppE linear peptide wherein the Cys side chain bears an acetamide adduct after treatment with IAA. Note that dehydration and thioether macrocyclization produce isomeric products, but the Cys side chain thiol is not involved in thioether formation for dehydrated peptide product. <u>The observation that an IAA adduct is not observed for the dehydrated/macrocyclized MppE product implies that the MppM-catalyzed modification is macrocyclization, and not dehydration</u>. The y-axes in panels A and B are scaled identically. (**C**) MS^2 fragmentation spectrum for the $[M+H]^{1+}$ parent ion (labeled by blue diamond) corresponding to the unmodified MppE core peptide with the Cys side chain bearing the IAA adduct.

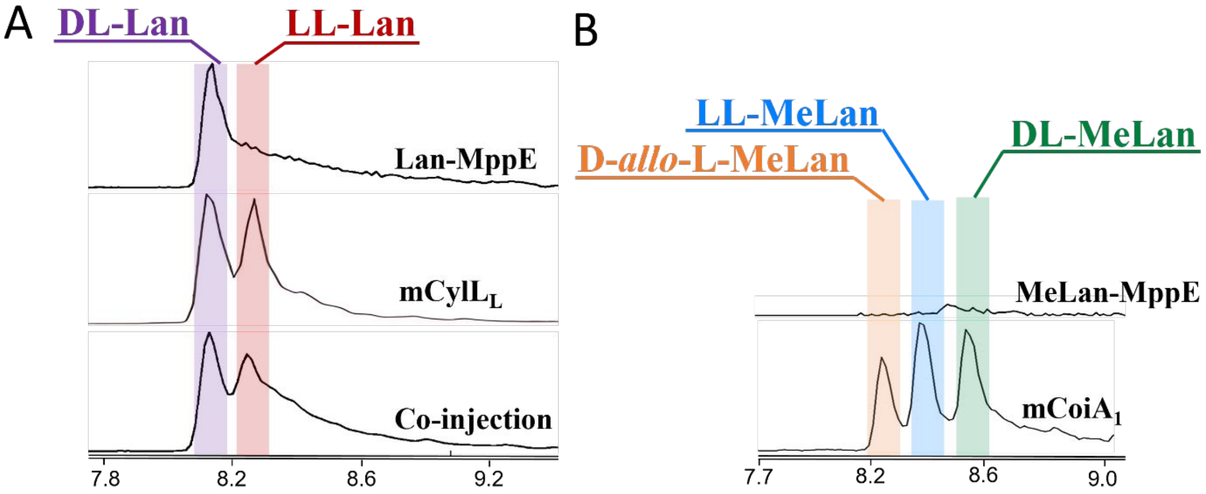

Figure S5: LC/MS analysis of L-FDLA-derivatized MppE core cyclic-ACWRWSG, Lan standard mCylL$_L$ and MeLan standard mCoiA$_1$. (**A**) EIC monitoring of Lan (L-DLA)$_2$ at $[M-H]^{1-}$ *m/z* = 795.2373 Da. Comparison of retention time and co-injection with standard establishes the configuration of the lanthionine ring in MppE as DL. (**B**) EIC monitoring of MeLan (L-DLA)$_2$ at $[M-H]^{1-}$ *m/z* = 809.2530 Da. Note that the modified MppE bears a lanthionine, and not a methyllanthionine ring.

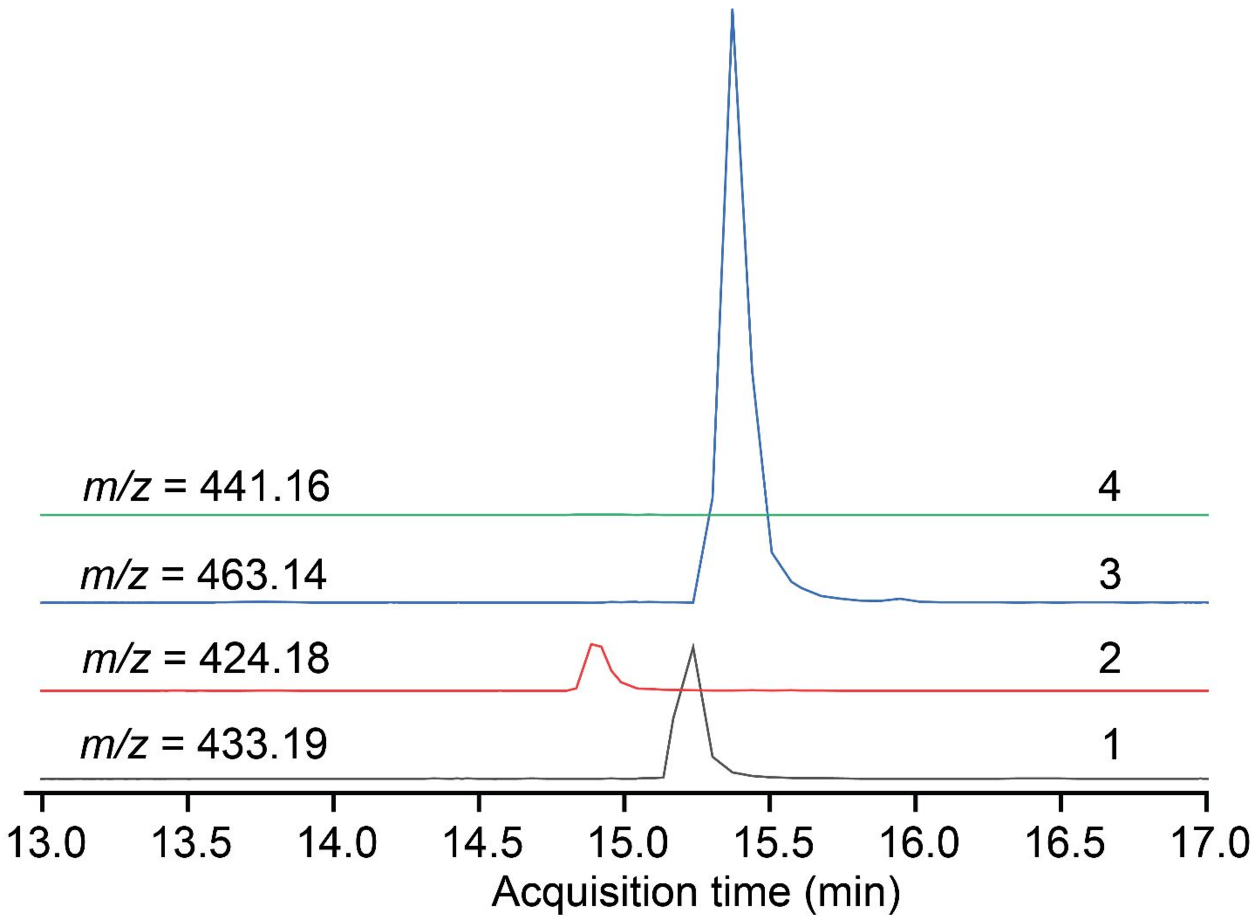

Figure S6: EICs for the $[M+2H]^{2+}$ parent ions corresponding to the unmodified linear MppE core peptide (**1**), macrocyclized MppE core peptide (**2**), macrocyclized and mono-brominated MppE core peptide (**3**), and hypothetical macrocyclized and mono-chlorinated MppE core peptide (**4**) when *mppE* was co-expressed with *mppM* and *mppI* in *E. coli*. Note that the y-axes for all four EICs are scaled identically such that they are indicative of relative peptide abundance. Even though *E. coli* growth media is replete with chloride, no chlorinated product was detected in this experiment (EIC **4**) which indicates that MppI is an obligate brominase. The MppE core peptides were excised from the full length MppE peptide using the peptidase LahT150.

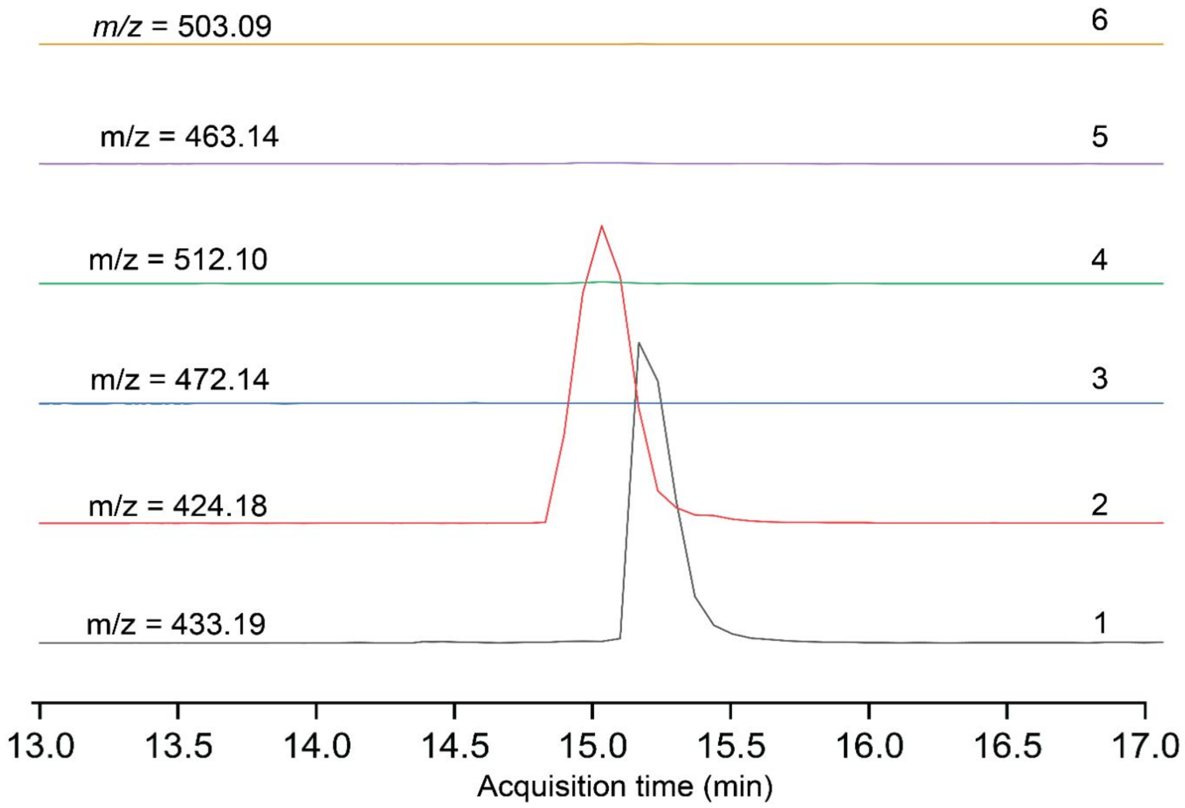

Figure S7: EICs for the $[M+2H]^{2+}$ parent ions corresponding to the unmodified linear MppE core peptide (**1**), macrocyclized MppE core peptide (**2**), mono-brominated linear MppE core peptide (**3**), di-brominated linear MppE core peptide (**4**), macrocyclized and mono-brominated MppE core peptide (**5**) macrocyclized and di-brominated MppE core peptide (**6**), when *mppE* was co-expressed with *mppM* and *mppI*-K73A in *E. coli.* The y-axes for all four EICs are scaled identically such that they are indicative of relative peptide abundance. Note that the K73A mutation abolishes production of any brominated peptides in the experiment.

A

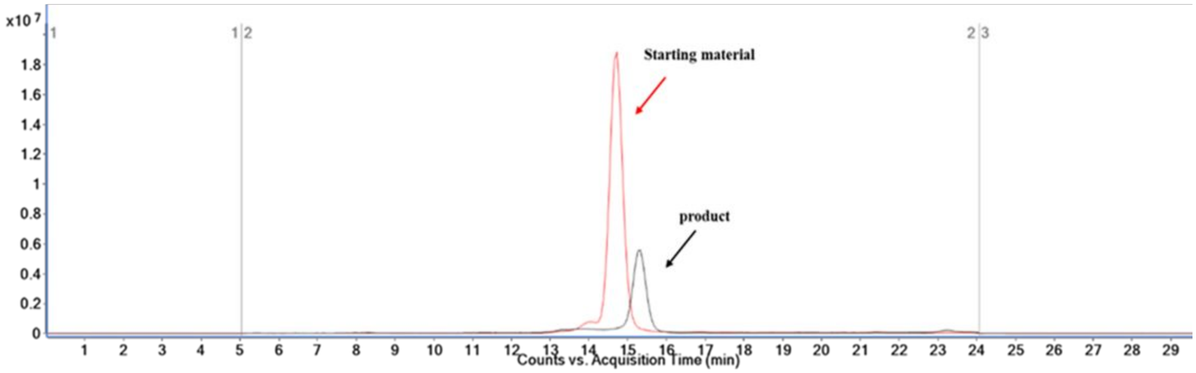

B

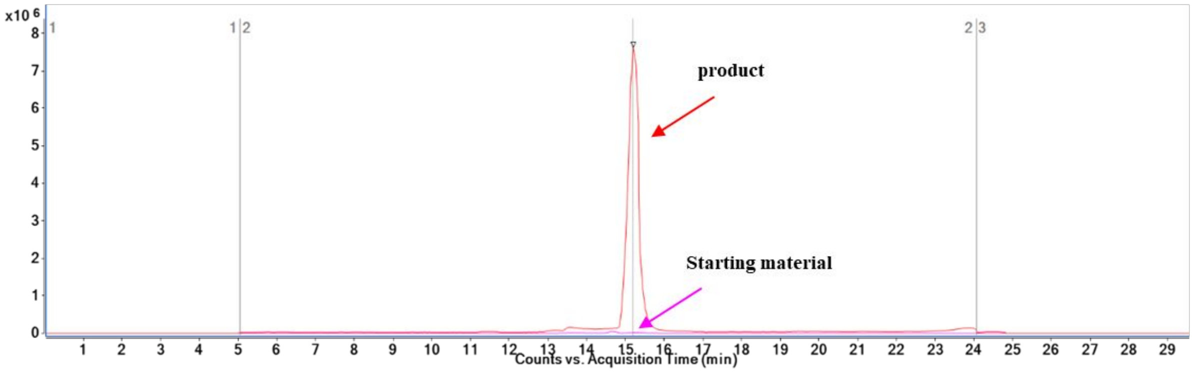

Figure S8: (**A**) EICs corresponding to the macrocyclic MppE W80F core peptide (labeled 'Starting material') and the macrocyclic mono-brominated MppE W80F core peptide (labeled 'product') detected after LahT150 digestion when *mppE*-W80F gene was co-expressed with *mppM* and *mppI* in *E. coli*. (**B**) EICs corresponding to the macrocyclic MppE W82F core peptide (labeled 'Starting material') and the macrocyclic mono-brominated MppE W82F core peptide (labeled 'product') detected after LahT150 digestion when *mppE*-W82F gene was co-expressed with *mppM* and *mppI* in *E. coli*. Note that the W80F mutation greatly reduces brominated product formation, while W82F mutation does not, leading us to posit that W80 is the site of bromination by MppI. When W80 is mutagenized to Phe, the promiscuous activity of MppI would brominate the W82 side chain indole, akin to the detection of dibrominated products illustrated in Figure S11 (*vide infra*).

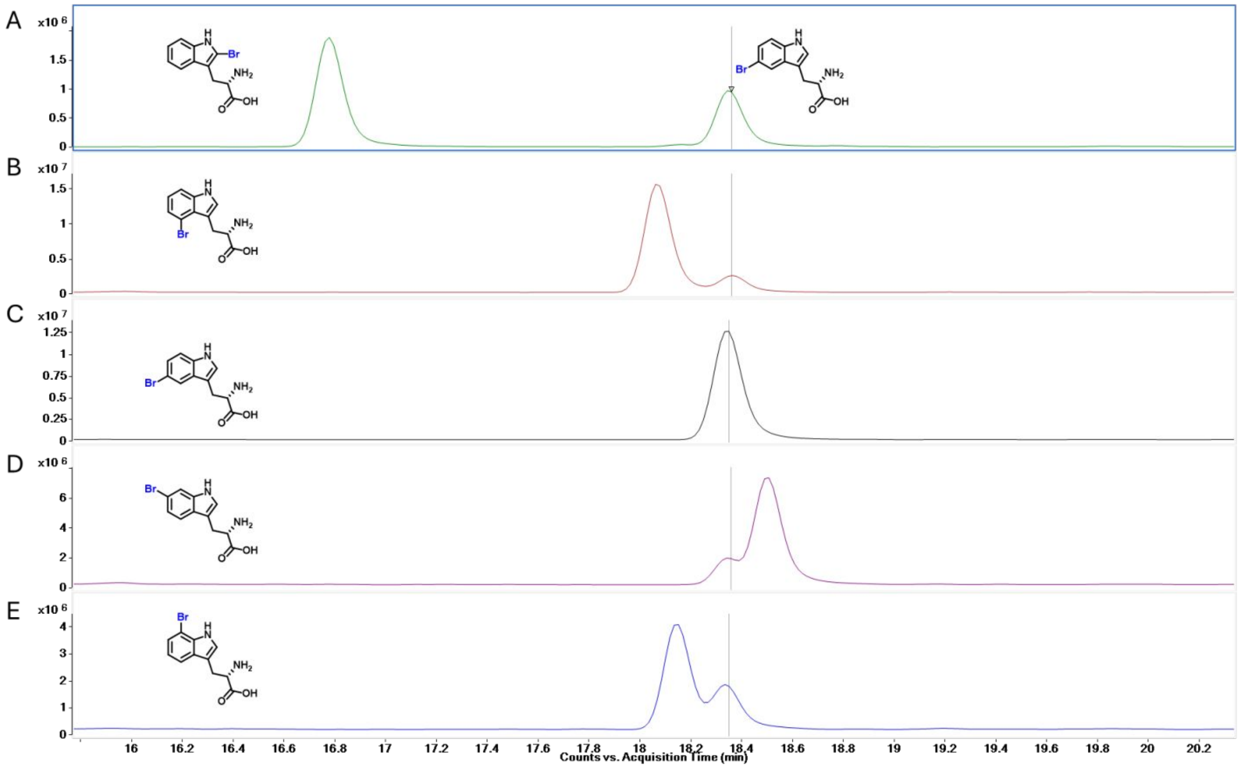

Figure S9: The carboxypeptidase digestion reaction for cyclic and brominated MppE core peptide was co-injected with (from top to bottom) standards for 2-, 4-, 5-, 6-, and 7-bromotryptophan. The presence of mono-brominated tryptophan was monitored by LC/MS using EICs for *m/z* 283.01±0.1 Da. The bromotryptophan residue excised from MppE co-eluted with the 5-bromotryptophan standard.

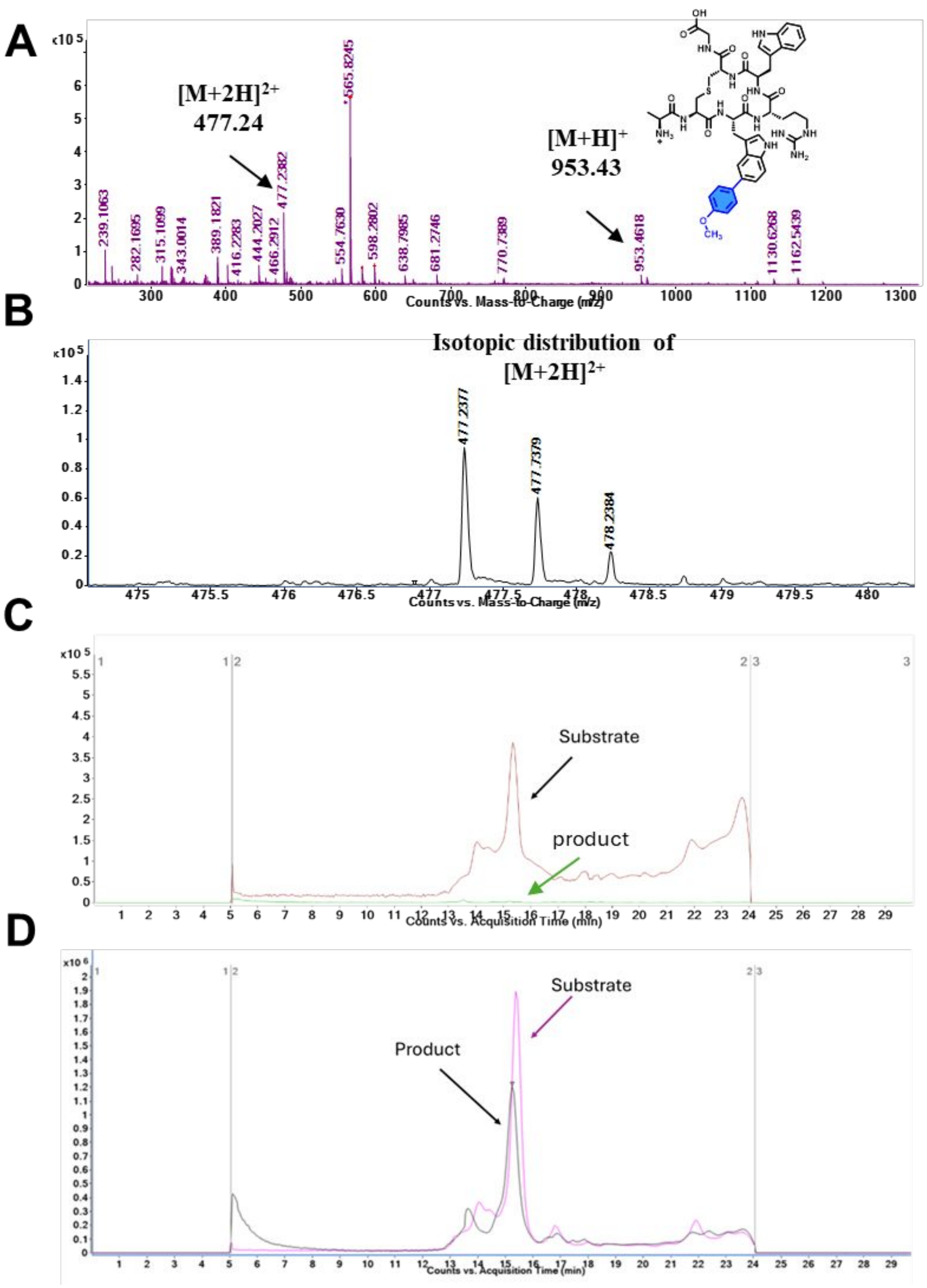

Figure S10: (**A**) MS^1 spectra and (**B**) the isotopic signature of the $[M+2H]^{2+}$ ions for the MppE core peptide with the methoxyphenyl moiety acylated to the Trp80 side chain indole via the Suzuki-Miyaura cross coupling (SMCC) reaction. (**C**) EICs demonstrating the relative abundances of the brominated substrate and the SMCC product in a reaction where the catalyst was omitted. (**D**) EICs demonstrating the relative abundances of the brominated substrate and the SMCC product in a reaction where all components were present. The area under these EICs was used to calculate the yield of the SMCC reaction.

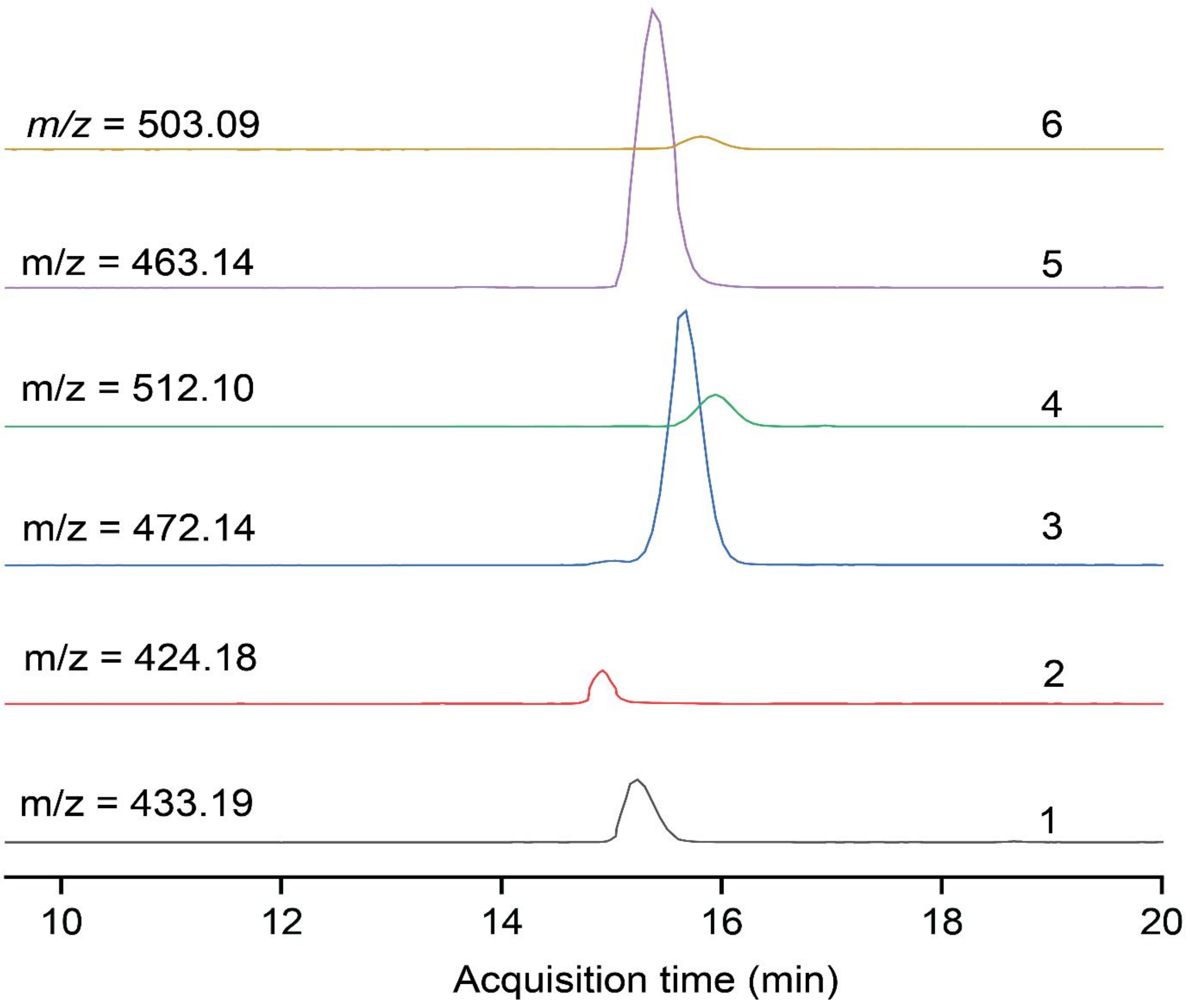

Figure S11: EICs for the $[M+2H]^{2+}$ parent ions corresponding to the unmodified linear MppE core peptide (**1**), macrocyclized MppE core peptide (**2**), mono-brominated linear MppE core peptide (**3**), di-brominated linear MppE core peptide (**4**), macrocyclized and mono-brominated MppE core peptide (**5**), and the macrocyclized and di-brominated MppE core peptide (**6**) when *mppE* was co-expressed with *mppM* and *mppI* in *E. coli*. The y-axes for all EICs are scaled identically such that they are indicative of relative peptide abundances. Note that the peaks in chromatograms **3** (relative to **5**) and **4** (relative to **6**) demonstrate the presence of linear brominated products wherein macrocyclization by MppM has been bypassed.

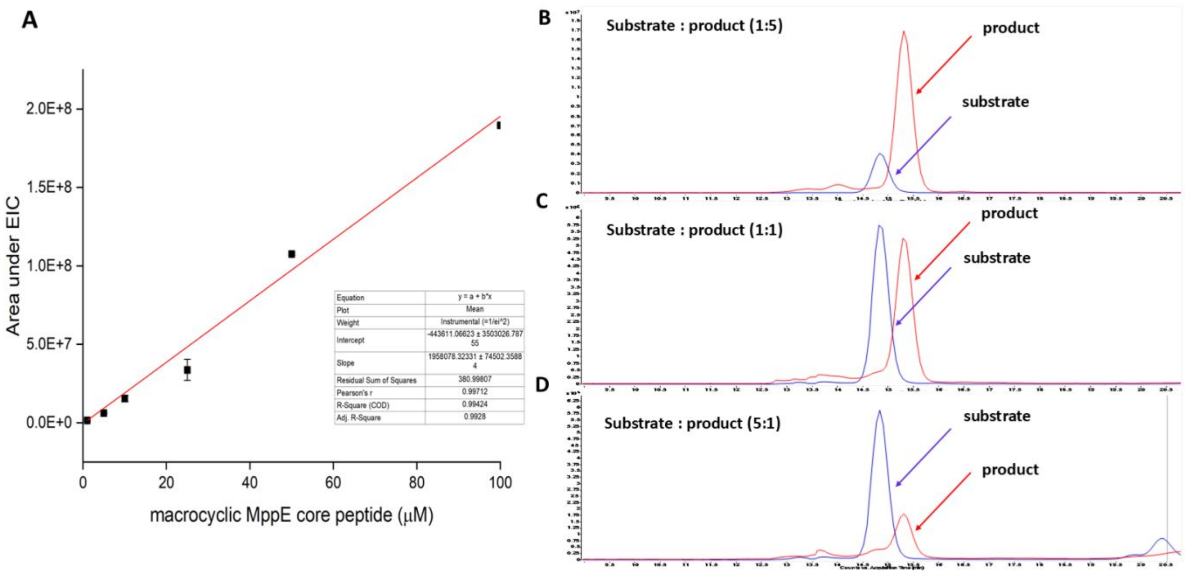

Figure S12: (**A**) Calibration curve between area under the EIC and the concentration of macrocyclic MppE core peptide detected by the mass spectrometer during typical LC/MS experiments described in this study. Mean and standard deviations from three independent injections are plotted. The concentration of precursor peptides in assays described in this study is 25 µM or less which implies that the substrates and products fall within the linear range of detection of the mass spectrometer. (**B**–**D**) Bromination does not alter the ionization of the macrocyclic MppE core peptide. The macrocyclic MppE core peptide (labeled "substrate"; EICs for the for the $[M+2H]^{2+}$ parent ions in blue) was mixed with the brominated macrocyclic MppE core peptide (labeled "product" EICs for the for the $[M+2H]^{2+}$ parent ions in red) in (**B**) 1:5 molar ratio, (**C**) 1:1 molar ratio, and (**D**) 5:1 molar ratio and analyzed by LC/MS. Higher molar abundance of the respective peptide was faithfully reproduced by the EICs. Mass spectrometry data were collected in the positive ionization mode. Chromatography was performed using Agilent Poroshell 120 2.7 µm C_{18} reversed-phase column (100×4.6 mm) at a flow rate of 0.3 mL/min using the following solvents for the mobile phase: solvent A: H_2O + 0.1% (v/v) formic acid; solvent B: MeCN + 0.1% (v/v) formic acid. The chromatography elution profile was as follows: 5% solvent B from 0 to 5 min, linear gradient to 100% solvent B from 5 to 18 min, 100% solvent B from 18 to 22 min, linear gradient to 5% solvent B from 22 to 24 min, and 5% solvent B from 24 to 30 min. Mass spectrometry data were acquired from between 5 min and 24 min. For MppE peptides, the $[M+2H]^{2+}$ parent ions dominated the MS^1 spectra. The area under the EICs for the $[M+2H]^{2+}$ MS^1 ions were used to quantify peptide amounts.

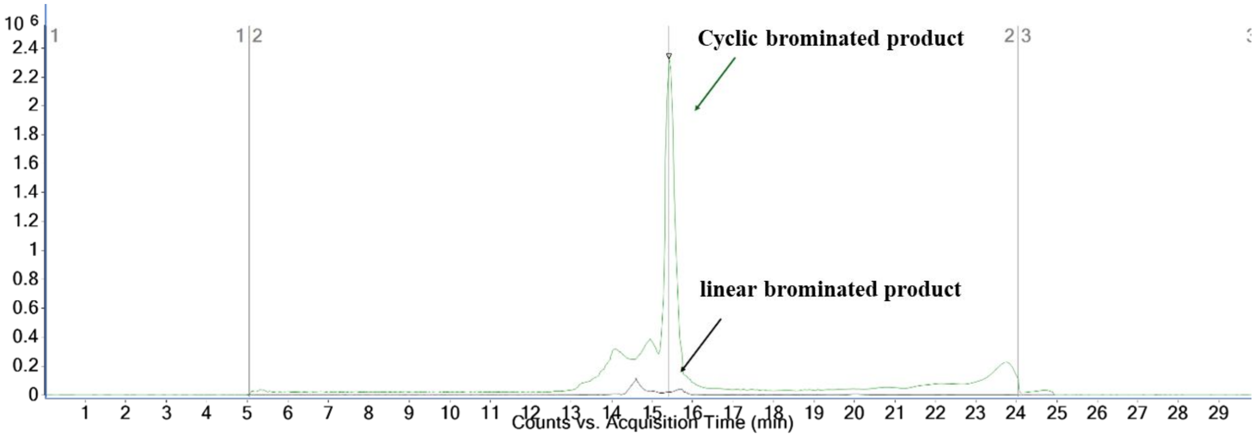

Figure S13: EICs denoting the relative abundance of the macrocyclic MppE mono-brominated product and the linear MppE mono-brominated product in a competition experiment wherein the macrocyclic MppE and linear MppE were provided in an equimolar ratio to MppI. Note that MppI prefers to brominate macrocyclic MppE peptide as inferred from higher abundance of the mono-brominated macrocyclic MppE product as compared to the abundance of the mono-brominated linear MppE product.

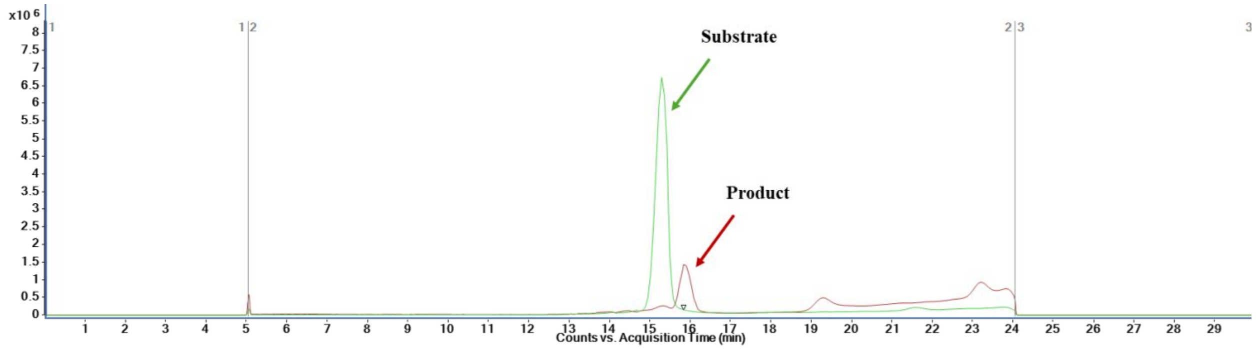

Figure S14: Representative EICs demonstrating the relative abundance of the starting material and the mono-brominated product when linear unmodified MppE was provided as the substrate to MppI in an *in vitro* bromination reaction.

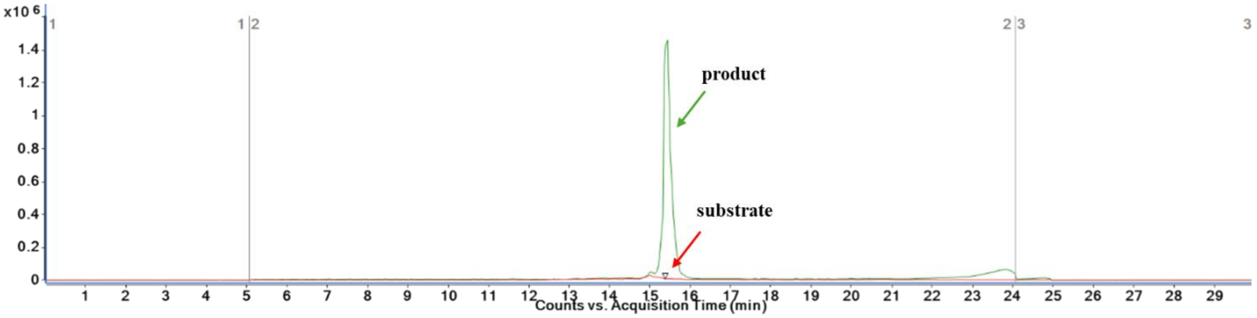

Figure S15: Representative EICs demonstrating the relative abundance of the starting material and the mono-brominated product when macrocyclic MppE was provided as the substrate to MppI in an *in vitro* bromination reaction.

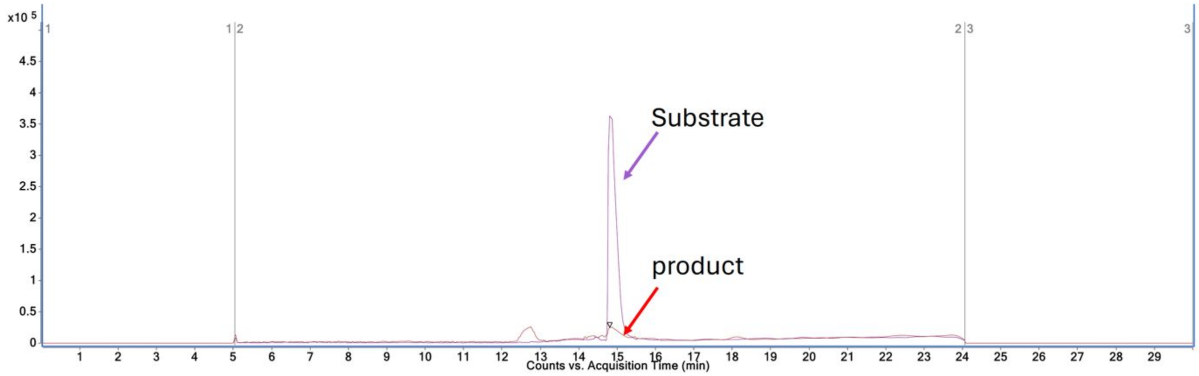

Figure S16: EICs demonstrating the relative abundance of the starting material and the mono-brominated product when macrocyclic MppE core without the leader peptide was provided as the substrate to MppI in an *in vitro* bromination reaction.

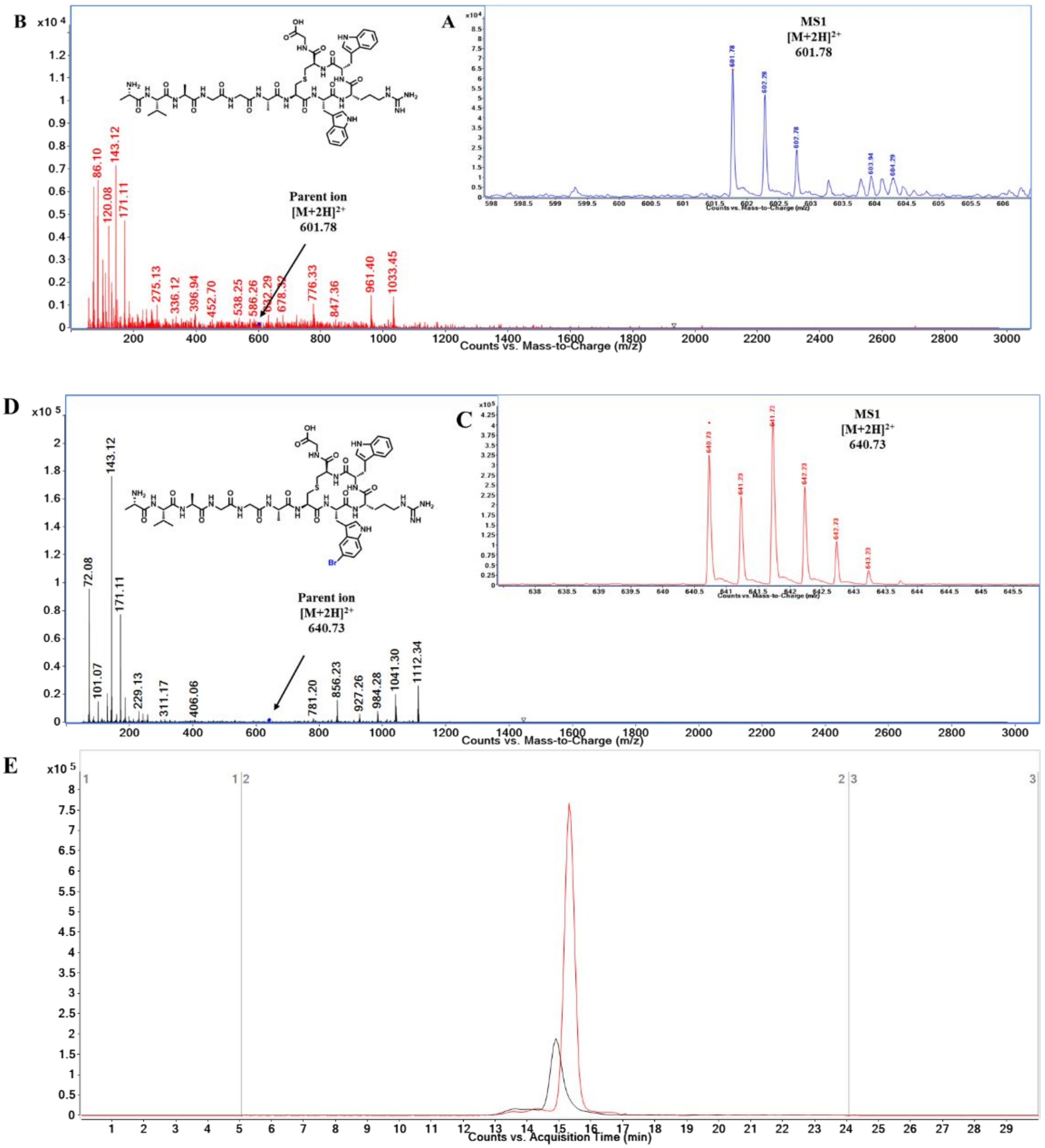

Figure S17: The L(X)$_4$L→A(X)$_4$A mutation compromises the LahT150 activity. Hence, the peptides here were digested with Glu-C rather than LahT150. (**A**) MS^1 and (**B**) MS^2 spectra of the substrate macrocyclic MppE L66A/L71A peptide after digestion with Glu-C. (**C**) MS^1 and (**D**) MS^2 spectra of the product macrocyclic mono-brominated MppE L66A/L71A peptide after digestion with Glu-C. (**E**) EIC (in black) for the leftover substrate and (in red) for the mono-brominated product demonstrating the relative substrate/product abundances for the MppI-catalyzed bromination of macrocyclic MppE L66A/L71A peptide.

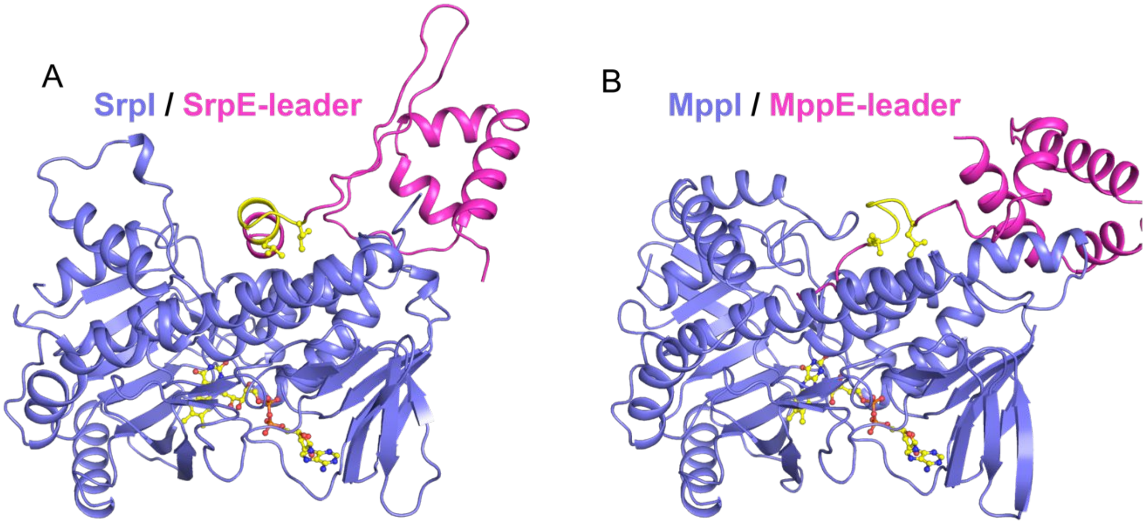

Figure S18: AlphaFold3-predicted models for (**A**) SrpE leader binding to SrpI and (**B**) MppE leader binding to MppI. The flavin-adenine dinucleotide (FAD) cofactor is shown in stick ball representation with carbon atoms colored yellow. The $L(X)_4L$ motif in the SrpE and MppE peptides is colored yellow with the Leu side chain atoms represented in stick-ball representation. The structure of the SrpE leader that is predicted by AlphaFold3 is in good agreement with the experimentally determined structure.[3]

SUPPLEMENTARY REFERENCES

1. Gerlt, J. A.; Bouvier, J. T.; Davidson, D. B.; Imker, H. J.; Sadkhin, B.; Slater, D. R.; Whalen, K. L., Enzyme Function Initiative-Enzyme Similarity Tool (EFI-EST): A web tool for generating protein sequence similarity networks. *Biochim Biophys Acta* **2015,** *1854*, 1019-37.

2. Shannon, P.; Markiel, A.; Ozier, O.; Baliga, N. S.; Wang, J. T.; Ramage, D.; Amin, N.; Schwikowski, B.; Ideker, T., Cytoscape: A Software Environment for Integrated Models of Biomolecular Interaction Networks. *Genome Research* **2003,** *13*, 2498-2504.

3. Nguyen, N. A.; Vidya, F. N. U.; Yennawar, N. H.; Wu, H.; McShan, A. C.; Agarwal, V., Disordered regions in proteusin peptides guide post-translational modification by a flavin-dependent RiPP brominase. *Nature Communications* **2024,** *15*, 1265.

4. Luo, Y.; Xu, S.; Frerk, A. M.; van der Donk, W. A., Facile Method for Determining Lanthipeptide Stereochemistry. *Analytical Chemistry* **2024,** *96*, 1767-1773.

5. Romney, D. K.; Murciano-Calles, J.; Wehrmüller, J. E.; Arnold, F. H., Unlocking Reactivity of TrpB: A General Biocatalytic Platform for Synthesis of Tryptophan Analogues. *Journal of the American Chemical Society* **2017,** *139*, 10769-10776.

6. Nguyen, N. A.; Lin, Z.; Mohanty, I.; Garg, N.; Schmidt, E. W.; Agarwal, V., An obligate peptidyl brominase underlies the discovery of highly distributed biosynthetic gene clusters in marine sponge microbiomes. *Journal of the American Chemical Society* **2021,** *143*, 10221-10231.
